# Advancements and sustainable strategies for the treatment and management of wastewaters from metallurgical industries: an overview

**DOI:** 10.1007/s11356-023-30891-0

**Published:** 2023-11-14

**Authors:** Michail Chalaris, Despina A. Gkika, Athanasia K. Tolkou, George Z. Kyzas

**Affiliations:** Hephaestus Laboratory, Department of Chemistry, International Hellenic University, Kavala, Greece

**Keywords:** Wastewater treatment, Metallurgical industries, Physical method, Chemical method, Biological method, Metallurgical slag

## Abstract

Metallurgy is pivotal for societal progress, yet it yields wastewater laden with hazardous compounds. Adhering to stringent environmental mandates, the scientific and industrial sectors are actively researching resilient treatment and disposal solutions for metallurgical effluents. The primary origins of organic pollutants within the metallurgical sector include processes such as coke quenching, steel rolling, solvent extraction, and electroplating. This article provides a detailed analysis of strategies for treating steel industry waste in wastewater treatment. Recent advancements in membrane technologies, adsorption, and various other processes for removing hazardous pollutants from steel industrial wastewater are comprehensively reviewed. The literature review reveals that advanced oxidation processes (AOPs) demonstrate superior effectiveness in eliminating persistent contaminants. However, the major challenges to their industrial-scale implementation are their cost and scalability. Additionally, it was discovered that employing a series of biological reactors instead of single-step biological processes enhances command over microbial communities and operating variables, thus boosting the efficacy of the treatment mechanism (e.g., achieving a chemical oxygen demand (COD) elimination rate of over 90%). This review seeks to conduct an in-depth examination of the current state of treating metallurgical wastewater, with a particular emphasis on strategies for pollutant removal. These pollutants exhibit distinct features influenced by the technologies and workflows unique to their respective processes, including factors such as their composition, physicochemical properties, and concentrations. Therefore, it is of utmost importance for customized treatment and disposal approaches, which are the central focus of this review. In this context, we will explore these methods, highlighting their advantages and characteristics.

**Supplementary Information:**

The online version contains supplementary material available at 10.1007/s11356-023-30891-0.

## Introduction

Metals play a crucial role in our society, and the metallurgical industry encompasses various activities such as mineral exploration, mining, selection, smelting, and rolling timber. Both ferrous and non-ferrous metallurgical sectors are primarily responsible for extracting and refining metals, with a growing focus on sustainable manufacturing practices that minimize environmental impact (Wu et al. 2017a). In Asian countries (China, India, etc.), the iron and steel industries play a vital role in fostering economic growth and progress (Zhao et al. 2021). However, alongside the production of metal products, waste in various forms is also created. Within the metallurgical industry, several kinds of waste are produced, including different types of dusts and sludge (Iluţiu-Varvara and Aciu 2022). These metallurgical slags contain hazardous heavy metals like lead, chromium, and cadmium that pose environmental risks (Rahou et al. 2022).

Water is extensively utilized in different stages of metallurgical processes, such as mining, coking, steelmaking, and electroplating. The production of steel, in particular, demands the use of huge quantities of water (Tong et al. 2018). Unfortunately, the industrial sector’s water demand has escalated considerably in recent decades, leading to the overuse of water resources to cover these needs (Srivastava et al. 2021). The metallurgical sector contributes substantially to the overall volume of industrial wastewater generated (Wu et al. 2017a).

The steel industry plays a pivotal role in global economic development. The global iron and steel market size was valued at USD 1,676.24 billion in 2022 and is expected to grow at a compound annual growth rate (CAGR) of 3.8% from 2023 to 2030 (Grand View Research n.d.) However, due to its extensive manufacturing output, it generates a substantial volume of wastewater. This wastewater originates from various processes within steel production, including pig iron smelting in blast furnaces, rolling mills, and Linz-Donawitz (LD) converters (Das et al. 2021a). Moreover, this wastewater is laden with a plethora of contaminants and toxins, posing significant threats to both the environment and public health. In general terms, it can be categorized as follows: (i) physical characteristics, these include factors such as temperature, color, and odor. (ii) Chemical characteristics, they encompass parameters like pH, chemical oxygen demand (COD), and heavy metals. (iii) Biological characteristics, this category comprises biochemical oxygen demand (BOD), as well as microbes and the oxygen required for nitrification and microbial populations (Zhuang et al. 2019). Wastewater contaminants may be organic, inorganic, or radioactive. The choice of pre- and post-treatment systems depends on the nature of these contaminants (Sathya et al. 2022). For a concise overview of the classification, main effluents and characteristics of wastewaters from iron and steel industries and non-ferrous metallurgy, please refer to Table 1.

**Table 1:** Classification, main effluents, and characteristics of wastewaters from the metallurgical industry

Water source	Main effluents	Types of wastewater	Wastewater characteristics
Iron and steel industrial wastewater	Heavy metals Aromatic compounds Surfactants Cyanides Fluorides (Sathya et al. 2022)	Process (Raw material system, coking, sintering and pelletizing, iron smelting, steelmaking and continuous casting, hot/cold rolling) (Wu et al. 2017b).	Physical characteristics Temperature, turbidity, total suspended solids, color, and odor (Zhuang et al. 2019)
		Integrated (Wu et al. 2017b).	Chemical characteristics Chemical oxygen demand (COD), total organic carbon (TOC), heavy metals, dissolved oxygen (DO), toxic substances, pH, phosphorus, sulfur, chlorides, and other trace elements.(Zhuang et al. 2019).
Non-ferrous metallurgical wastewater	Copper, lead, and zinc) and xanthate (S. Meng et al. 2022) Arsenic and cadmium (Qi et al. 2023)	Heavy, light or rare earth non-ferrous metallurgical wastewater (P. Wu et al. 2017b).	Biological characteristics Biochemical oxygen demand (BOD), as well as microbes like bacteria, viruses, parasites, and the oxygen required for nitrification and microbial populations (Zhuang et al. 2019).
		Acidic, alkali, heavy metal, fluoride, cyanide, oil-containing or radioactive wastewater (Wu et al. 2017b)	

In non-ferrous metallurgy, a wide range of complexities arise due to variations in the composition and physicochemical properties of deposits, minerals, and final products (Wu et al. 2017b). Therefore, wastewaters generated during mining, extraction, purification, and fabrication in non-ferrous metallurgy are highly specific to the metal speciation involved. These wastewater streams primarily encompass fluorine and sulfur-containing effluents in heavy metal industries, acidic/alkaline wastewater in rare earth hydrometallurgy, high-concentration ammonia nitrogen (NH3-N) wastewater, and cyanide-laden wastewater originating from the noble metal industry (Cornelis et al. 2008). Additionally, there are electroplating wastewater and organic chloride wastewater, albeit in relatively smaller quantities, which still pose hazards (Agrawal and Sahu 2009).

One common trait among waste streams linked to metallurgical processes is their intricate composition and properties, stemming from the constitution of raw materials and the practice of amalgamating discharges from multiple stages. For example, wastewater emerging from rare earth metallurgy contains radioactive elements due to their paragenetic relationship with rare earth metals. Furthermore, certain metallurgical wastewaters can exhibit high levels of toxicity and hazard, primarily due to the chemical nature of raw materials, chemical agents, and/or byproducts involved in the production process. Among the most prevalent and harmful metallurgical contaminants are heavy metals, organic compounds, cyanide, and acids (Wu et al. 2017b).

A notable characteristic of waste streams originating from metallurgical processes is their complex composition and properties due to the nature of the raw materials involved and the integration of discharges from various process steps (Wei et al. 2012). Furthermore, certain metallurgical wastewaters can be extremely toxic and dangerous, as a result of the nature of the raw materials, the agents used, or side products generated during production (Sun et al. 2019). Unlike ferrous metallurgy, there is no universally accepted classification for non-ferrous metallurgy wastewaters, owing to the extensive variations in composition and physicochemical properties of deposits, minerals, and final products (Wu et al. 2017a). Therefore, it is imperative to treat industrial effluents appropriately before their discharge to counter any potential health or environmental hazards (Elsheikh et al. 2022).

The typical wastewater treatment process consists of multiple tiers is also employed for treating wastewater stemming from the steel industry (Pal 2017). Nonetheless, recent progress has unveiled the efficacy of diverse methods in eliminating contaminants from wastewater generated by the steel industry. These methods include — among others — membrane filtration, adsorption, and advanced oxidation processes (AOPs) (Ken and Sinha 2021). AOPs, such as photodegradation (Biswas et al. 2020), electrochemical oxidation (Ken and Sinha 2021), and the Fenton process (Teixeira et al. 2015), have demonstrated successful elimination of typical pollutants (BOD, COD), as well as other pollutants like cyanide (*CN*^−^), heavy metals and phenol. Additionally, the adsorption process has proven to be capable of removing metallic ions to an extent exceeding 99% (Abd El-Azim et al. 2019).

The steel industry operates as a complex system with multiple manufacturing units that generate intricate wastewater, posing challenges in its treatment. This review provides a timely assessment of the suitability of different typically used as well as growing technologies for treating wastewater in the steel industry. It delves into a comprehensive analysis of integrating and hybridizing different techniques, considering associated costs to optimize processes and formulate sustainable strategies for managing effluent. Furthermore, the review emphasizes the importance of practices for the sustainable utilization of waste and explores opportunities for effluent recycling and reuse. By suggesting greener and more effective systems for treating and managing effluents, this review addresses the increasing environmental concerns. While steel and iron industries have received less attention due to the presence of numerous effluent-generating units with varying characteristics and volumes, the primary aim of this review is to gather the segmented information from literature in an effort to examine, propose, and discuss the processes employed within the metallurgical industry, the volume of effluents generated, as well as the latest cutting-edge theoretical and scientific advancements in treatment techniques designed for the removal of these substances. Additionally, this article will address the challenges and future prospects associated with these developments. The novelty of this study is centered on its objective to reevaluate existing literature and offer contemporary, readily comparable insights into advancements and sustainable approaches for handling and treating wastewater generated by metallurgical industries, with a specific focus on elucidating the factors that impact these processes.

## Tannery and flue gas desulfurization at the forefront of environmental pollution

The metallurgical processes generate substantial volumes of wastewater, contributing to approximately 730 million tons per year of effluent from the nonferrous metallurgical industry in China. Consequently, wastewater from the nonferrous metallurgical sector represents a significant source of heavy metals. Tannery sludge poses a particular challenge due to its composition, containing a wide range of undesirable substances, including organics, inorganics, and metallic elements, with a particularly high concentration of chromium (Wang et al. 2021). In the chrome tanning process, a total of 276 chemicals and 14 heavy metals are employed, playing a prominent role in water pollution. Given that most tannery industry processes are wet processes, generating substantial volumes of liquid waste, the effluents produced often fall far short of acceptable levels due to the heavy burden of pollutants, including chromium, chlorides, sodium, dissolved solids, BOD, COD, nitrogen, and suspended solids. Chromium holds particular significance in various metallurgical applications (Mohammed and Sahu 2019).

The iron and steel industry plays a dual role in our country, serving as both a critical foundational sector and a significant contributor to high energy consumption, elevated emissions, and increased environmental burdens (Meng 2020). This industry not only consumes vast quantities of raw materials but also energy resources, while concurrently emitting pollutants (Meng 2020). The generation of air pollutants within the iron and steel sector arises from multiple processes, including the blast furnace, coke oven, sintering, pellet production, and iron alloy furnaces (Zou et al. 2020). Among these processes, sintering holds particular importance in iron and steel production (Cheng et al. 2016). Notably, the sintering process is responsible for 40% of the total emissions generated during steel production (Zhang et al. 2020a, 2020b). Sintering flue gas exhibits complex characteristics, encompassing a diverse composition, a wide temperature range, high oxygen content, and elevated moisture levels. Consequently, the treatment and purification of sintering flue gas pose significant challenges in the iron and steel industry (Qian et al. 2018).

The swift development of flue gas treatment technology has been driven by stringent pollutant emission standards. Flue gas desulfurization (FGD) technology encompasses wet, dry, and semi-dry FGD methods (Song et al. 2020a, 2020b). Among these, the wet process stands out as the most widely employed and mature technology for mitigating SO_2_ emissions within the iron and steel industry (Koralegedara et al. 2019). In particular, wet flue gas desulfurization (WFGD) systems are extensively utilized in coal-fired power plants. During the WFGD process, FGD wastewater is routinely discharged from the desulfurization tower to control the chloride (Cl−) concentration within the desulfurization slurry. The accumulation of Cl− within the WFGD system can lead to corrosion of pipes and equipment by breaching the passive metal film (Liu et al. 2023). Conventional coal-fired power plants have experienced extensive use to meet the growing demand for electricity consumption (Koplitz et al. 2017). Consequently, flue gas desulfurization (FGD) systems have become essential for achieving ultraclean flue gas treatment in these conventional coal-fired power plants to protect the atmospheric environment (Cui et al. 2018). Periodic discharge of FGD wastewater from the system is essential to maintain the chloride (Cl−) concentration, preventing equipment corrosion and ensuring gypsum quality. Given the urgent need for more effective zero liquid discharge (ZLD) management strategies in conventional coal-fired power plants, interest has surged in desalination and Cl− removal from FGD wastewater (Fu et al. 2018; Han et al. 2019; Ye et al. 2021).

Numerous Cl− removal technologies have been developed, including evaporation crystallization, electrolysis, adsorption, ion exchange, reverse osmosis, and more (Ye et al. 2021). However, when applied to ZLD of FGD wastewater, these technologies often demand prolonged processes, substantial upfront investments, and high operational costs due to the intricate characteristics and strong scaling tendencies of FGD wastewater (Fu et al. 2018). Chemical precipitation methods involving copper slag or silver salt addition offer a simple, rapid, and efficient dechlorination process. However, these precipitants are often deemed prohibitively expensive for industrial applications (Sun et al. 2021). In this context, Friedel’s salt precipitation (FSP) has emerged as a highly efficient chloride removal method, garnering significant attention for ZLD of flue gas desulfurization (FGD) wastewater. The FSP method achieves Cl− removal through the formation of Friedel’s salt [Ca2Al(OH)6Cl·2H2O] (FS) via a reaction between lime (CaO) and an aluminum (Al) compound, typically aluminate (Abdel-Wahab and Batchelor 2002).

## Wastewater treatment strategies

Typical wastewater treatments comprise generally of an assortment of physicochemical and/or biological processes (Crini and Lichtfouse 2019). The physical processes are useful in extracting solids from wastewater, most often via screens and filters (Yenkie 2019). Physical treatments use physical effects without altering the composition of the wastewater. The wastewater does not affect the chemical characteristics of the contaminants, but only isolates the contaminants from water. Such approaches include the use of natural forces (gravity, van der Waals forces, etc.) and physical barriers to extract the pollutants (Li et al. 2020). Chemical processes are usually paired with physical processes to extract more complicated contaminants (Yenkie 2019). The chemical treatment of wastewater can have various effects, such as the generation of insoluble solids and gases, the formulation of biodegradable compounds from non-biodegradable ones and the destruction or deactivation of chelating agents that can efficiently remove substances from wastewater (Li 2020).

Several critical factors (Shebl 2023) must be taken into consideration when eliminating heavy metals from industrial wastewater:

- Type and concentration of heavy metals: The specific heavy metals present in the wastewater, along with their concentrations, dictate the most suitable treatment approach. Certain methods excel at removing particular heavy metals, necessitating a thorough analysis of the wastewater to determine the optimal strategy.
- Environmental impact: When disposing of heavy metal-containing waste generated during the treatment process, proper measures should be in place. This may entail secure storage or approved disposal through a licensed waste management facility. Additionally, treatment methods might have adverse environmental effects, such as the production of harmful byproducts or high energy consumption.
- Cost and feasibility: Assessing the cost and feasibility of the chosen treatment method is essential. Some methods may be more expensive or logistically complex to implement, demanding a careful evaluation of the costs versus the benefits of each option.
- Effectiveness: The effectiveness of the treatment method in reducing heavy metal concentrations to acceptable levels is of paramount importance. It is crucial to select a method capable of efficiently diminishing heavy metal concentrations in wastewater.
- Health and safety: Prioritizing the health and safety of personnel involved in the treatment process is critical. Appropriate safety measures must be in place to shield workers from exposure to hazardous materials.

Various treatments are available for removing heavy metals from water and other substances.

Adsorption is considered one of the most effective methods for separating diluted contaminants, offering opportunities for the recovery, reuse, and recycling of adsorbent materials (Senthil Kumar et al. 2019). While adsorption is not a recent technique for contaminant removal, it has firmly established itself as a globally recognized method due to challenges associated with the disposal and regeneration of adsorbents (Ngueagni et al. 2020). Thanks to its ease of operation, versatility in handling various substances, and its tolerance for hazardous chemicals, adsorption is widely regarded as one of the most efficient and dependable wastewater treatment methods (Gunasundari and Senthil Kumar 2017). Diverse nanocomposites have been employed as adsorbents for removing emerging contaminants from industrial effluent (Sophia and Lima 2018). Adsorption can be divided into two categories: physical adsorption (physisorption) and chemical adsorption (chemisorption). In physisorption, weak van der Waals forces are responsible for the attraction between molecules and the solid surface. This process is reversible and typically occurs at or near the critical temperature of the adsorbed substance. Chemisorption, on the other hand, involves chemical bonding between the adsorbed molecules and the solid surface, resulting in a monolayer of adsorbate molecules. Unlike physisorption, chemisorption forms a single molecular layer on the solid surface, and the adsorbates are strongly bound, making them less easily removed (Krishnan et al. 2017). Physical adsorption is characterized by a decrease in free energy and entropy, making it an exothermic process (Chai et al. 2021). Charged pollutants tend to adsorb onto oppositely charged adsorbents through electrostatic attraction. Metrics commonly used to understand the adsorption of metals by various materials include the Freundlich adsorption isotherm (KF) and Langmuir’s maximum adsorption capacity (qmax) (Chai et al. 2021).

**Fig 1:**
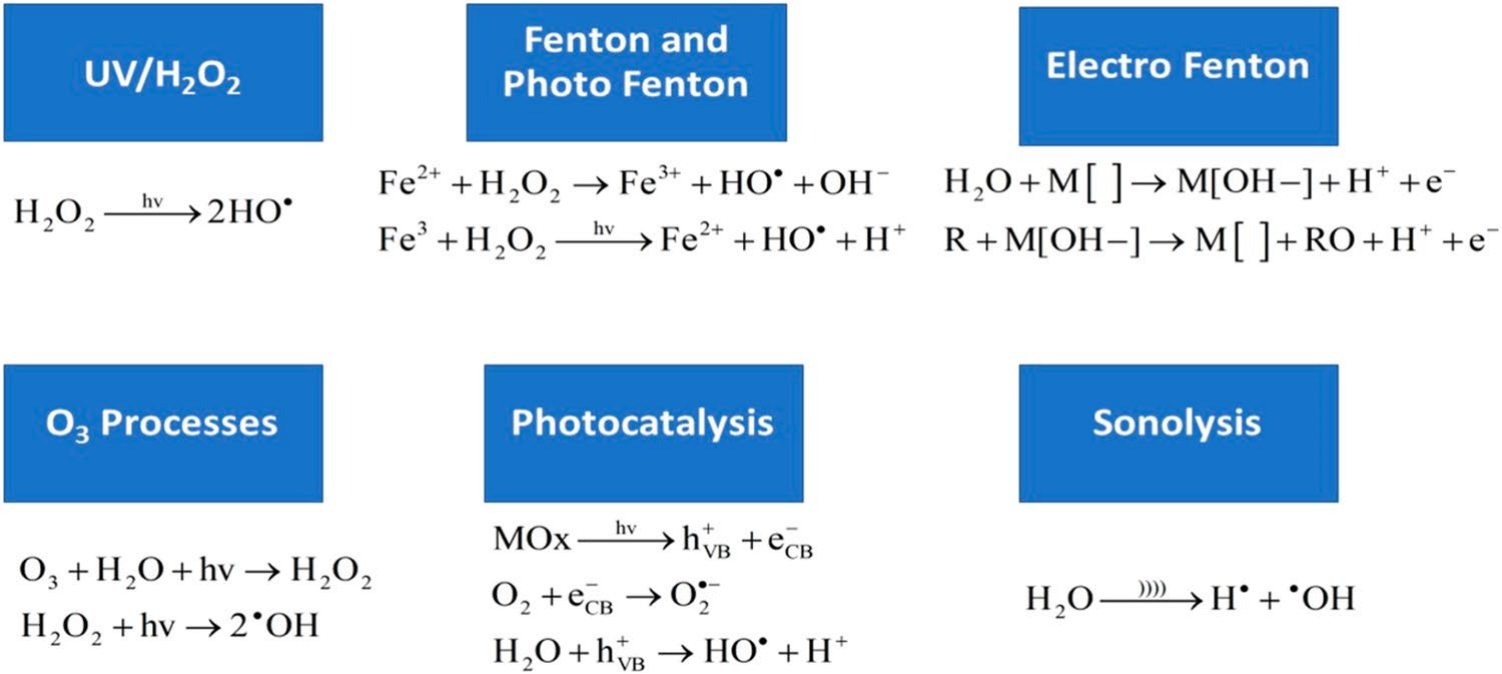
Overview of the mechanism in AOP (Pandis et al. 2022)

Advanced oxidation methods (Fig. 1) represent innovative approaches to pollutant removal, offering several advantages over conventional techniques. While biological oxidation has its merits, some complex compounds remain challenging to eliminate. Advanced oxidation processes can be integrated before or after biological treatments (Rathi et al. 2021). These processes encompass various methods, such as sonolysis, Fenton’s reaction, ozonation, UV radiation, photocatalysis, microwave radiation, and electrolysis followed by sonolysis (Pavithra et al. 2019). Advanced oxidation processes are highly effective in water and wastewater management. Hydroxyl radicals, which are potent oxidizing agents, play a key role in these processes and are produced primarily through ultraviolet (UV) light exposure. Different methods can be employed to initiate the generation of hydroxyl radicals (Rathi et al. 2021).

### UV/H_2_O_2_ (ultraviolet/hydrogen peroxide processes)

When UV light is employed in conjunction with hydrogen peroxide, it can efficiently produce hydroxyl radicals. Furthermore, UV light serves as a naturally occurring disinfectant for numerous organic contaminants and wastewater treatment systems. Extensive research has been conducted to refine the underlying chemistry of this procedure. It may be worthwhile to contemplate substituting costly hydrogen peroxide with alternative chemicals, such as chlorine, particularly under conditions of low pH and minimal UV exposure. Numerous variables impact the efficacy of these reactions, including the concentration of hydrogen peroxide, the origin of the UV radiation, and the physicochemical characteristics of the fluid involved in the oxidation process (Pandis et al. 2022).

### Fenton and photo-Fenton

To effectively address recalcitrant and substantial contaminants, UV radiation must be employed in conjunction with more potent agents. Fenton processes are favored due to their utilization of economical and less perilous substances, simpler equipment, and the environmentally impactful recycling of reactants in a cyclical manner. The Fenton process encompasses various stages, including pH adjustment, the primary oxidation reaction, neutralization, and precipitation. Notably, the photo-Fenton process has demonstrated noteworthy enhancements in reducing color and chemical oxygen demand (COD) when compared to the conventional Fenton process. The efficiency of these processes is influenced by parameters such as pH, hydrogen peroxide concentration, UV radiation duration, and the dosage of iron sulfate (Pandis et al. 2022).

### Electro-Fenton

Electrochemical methods have gained attention for treating hazardous wastes. Anodic oxidation is typically responsible for the destruction of organic and toxic pollutants found in such wastes, including phenols and pesticides, generating oxidants like hydroxyl radicals and ozone (Arapoglou et al. 2003).

### Ozone-based (O_3_) processes

Involving ozone in aqueous reactions can produce various unwanted byproducts due to its high redox potential. These processes can be categorized as ozone/hydrogen peroxide and ozone/UV, with the former being more common in wastewater treatment due to enhanced hydroxyl radical production through H_2_O_2_ dissociation. Ozone-based processes generate powerful oxidants but can produce stable byproducts, including genotoxic compounds like formaldehyde. However, ozone has limited solubility in water, which presents challenges related to mass transfer at gas–liquid interfaces and self-decomposition. It is often used as a pretreatment step in wastewater treatment (Pandis et al. 2022; Wang et al. 2020).

### Photocatalysis

When semiconductor materials come into contact with ultraviolet or visible light, they initiate the formation of surface-activated areas that promote redox reactions, leading to the production of radicals such as HO• and O•−2. This phenomenon, referred to as photocatalysis, depends on the stimulation of semiconductor materials by photons, which results in the creation of electron-hole pairs. The band-gap energy of these semiconductor materials serves as a measure of their capability to undergo this excitation and trigger redox reactions (Vaitsis et al. 2020).

### Sonolysis (ultrasound radiation)

Sonochemistry utilizes ultrasound to promote or modify chemical reactions. Ultrasonic treatment induces various chemical and physical effects, making it suitable for material synthesis and modification. Ultrasound generates cavitation phenomena, where bubbles form, implode, and create high-energy conditions that drive chemical reactions. These localized conditions lead to alternative chemical reaction pathways with high product yields, all without significant temperature increases in the surrounding liquid medium (Sakkas et al. 2014).

### Constructed wetlands

Constructed wetlands are cost-effective wastewater treatment solutions capable of removing or reducing various waterborne pollutants, particularly pharmaceuticals and personal care products (Ding et al. 2017). In recent years, there has been growing interest in the removal of emerging organic pollutants by constructed wetlands. Environmentally friendly engineering systems, including free water surface (FWS) CW, horizontal subsurface flow (HSF) CW, and vertical subsurface flow (VSF) CW, have been designed for pollutant removal from contaminated water. Single-type CWs have been studied and implemented for many years. Integrated CW systems, often referred to as hybrid systems, combine two or more types of CWs to leverage their individual strengths and complement each other. A significant challenge in applying CWs is assessing whether the treatment performance of long-term CW operation can consistently meet design requirements, predicting the long-term effectiveness of CWs remains challenging due to variations in wetland construction conditions, plant species, water quality, and operational methods. Another essential aspect of CW applications is understanding the relationships between pollutant removal rates and influencing factors, such as temperature, hydraulic load, influent loads, and plant species. However, existing literature provides inconsistent information on these relationships (Wu et al. 2017).

### Membrane technology

Membrane technology is a physical method used to remove emerging contaminants from aquatic systems. Membranes are composed of materials with specific filtering properties, including surface charge, pore size, and hydrophobicity, allowing them to remove suspended contaminants. Membrane filtration includes ultrafiltration (UF), nanofiltration (NF), microfiltration (MF), forward osmosis (FO), and reverse osmosis (RO). Ultrafiltration operates at low pressure to remove colloidal, suspended, or dissolved pollutants, depending on the membrane and pollutant type. Microfiltration features pore sizes ranging from 0.1 to 10 μm and is typically operated at atmospheric pressure but may not effectively remove contaminants larger than 1 μm. Reverse osmosis and forward osmosis rely on osmotic pressure gradients and semi-permeable membranes to efficiently remove dissolved particles up to 1 nm in size from water. Nanofiltration membranes have small pore size ranges from 1 to 10 nm and exhibit high efficacy in removing emerging contaminants based on membrane type and contaminant characteristics (Mahmood et al. 2022).

### Electrocoagulation-electroflotation technology

Electrocoagulation-electroflotation (ECF) technology is a treatment process that utilizes electrical current to flocculate and treat contaminants without requiring the addition of coagulants. It involves pairs of metal sheets known as electrodes, arranged as anodes and cathodes. Electrocoagulation relies on electrochemistry principles, with the cathode undergoing oxidation (losing electrons) and the water undergoing reduction (gaining electrons), improving wastewater treatment, when the cathode electrode contacts wastewater, metal is released into the apparatus. This process neutralizes particulates by forming hydroxide complexes, leading to the agglomeration of particulates at the bottom of the tank, which can be removed through filtration. In an electrocoagulation-flotation apparatus, particulates instead float to the tank’s top due to hydrogen bubble formation generated at the anode. These floated particulates can be skimmed from the tank’s surface (Butler et al. 2011).

#### Pros constraints and main characeristics of wastewater treatments

It is important to recognize that each of the available options possesses distinct advantages and limitations, both in terms of cost and their effectiveness, suitability, and environmental impact, as highlighted by (Crini and Lichtfouse 2019). In view of the aforementioned considerations, it is reasonable to conclude that no single approach is universally suitable for achieving effective treatment. To address this challenge, a combination of various processes is needed. Table 2 provides a breakdown of their respective advantages and limitations.

**Table 2:** Pros constraints and main characeristics of wastewater treatments

Technology	Main characteristics	Pros	Cons
Adsorption	Nondestructive process Use of a solid material (Crini and Lichtfouse 2019)	Adsorption has a versatile range of uses in eliminating various contaminants, such as Cr, CN−, Mn, F, and more, from effluents produced by the steel industry. Leveraging waste by-products generated at various stages of steel production for the creation of cost-effective adsorbents offers a promising solution to address the challenge of managing sludge disposal (Rawat et al. 2023).	Adsorption relies on pH conditions, requires extended treatment durations, and experiences a gradual decline in adsorption capacity as the number of cycles increases. Additionally, regenerating adsorbents demands a source of either steam or vacuum (Das, Mondal, et al. 2021).
Electrocoagulation	Electrolysis (Crini and Lichtfouse 2019)	The electrocoagulation process is widely embraced because of its uncomplicated installation and maintenance system, economical operational costs, minimal sludge production, and its compatibility with other treatment methods like ultrasonic, microwave, and ozone treatments (Ebba et al. 2021)	The primary factor that exerts influence on the electrocoagulation process is the current density. Therefore, optimizing current density is essential to achieve efficient and cost-effective wastewater treatment (Rawat et al. 2023).
Photo-degradation and UV irradiation	Destruction by combustion (Crini and Lichtfouse 2019)	Robust radical-driven reactions, rapid contaminant removal, thorough degradation of pollutants, recycling capabilities, and harnessing visible light (Majumder et al. 2021) .	One drawback of the method is its reliance on UV light for activation (Aziz et al. 2016).
Ozonation	Use of an oxidant (Crini and Lichtfouse 2019)	No chemicals are required. Effective removal of a broad spectrum of microorganisms, both organic and inorganic chemicals. No necessity to alter pH or temperature. Enhanced germicidal efficiency (Guo et al. 2019)	The phenomenon of ozone reacting with diverse inorganic and organic compounds within the effluent is characterized by either direct ozone attack or an indirect process involving the generation of hydroxyl radicals during ozone decomposition (Das, Mondal, et al. 2021)
Flocculation	Uptake of the pollutants and separation of the products formed (Crini and Lichtfouse 2019)	Straightforward procedure. Incorporated physicochemical approach. Numerous chemicals are already available commercially. Minimal initial investment. Effective settling and dewatering of sludge. Substantial decrease in chemical and biochemical oxygen demands (Crini and Lichtfouse 2019).	Necessitates the addition of non-recyclable materials. Demands constant monitoring of effluent pH levels. Leads to increased sludge generation, necessitating additional management, treatment, and costs. Inefficient for the removal of arsenic (Crini and Lichtfouse 2019).
Precipitation	Uptake of the pollutants and separation of the products formed (Crini and Lichtfouse 2019)	Chemical precipitation is acknowledged as the most efficient approach for eliminating heavy metals from wastewater. It finds extensive application in industries due to its cost-effectiveness and straightforward operational procedures (Yadav et al. 2019)	The generation of sizable sludge volumes can result in challenges related to dewatering and disposal. Additionally, the amphoteric nature of metal hydroxide precipitation can be affected by the presence of complexing agents (Qasem et al. 2021).
UltraFiltration	Nondestructive separation Semipermeable barrier (Crini and Lichtfouse 2019)	It can be efficiently delivered via micro-pores evenly distributed across the membrane’s surface. With its uncomplicated design, high level of automation, user-friendly operation, and small footprint in iron and steel wastewater treatment processes, it offers significant benefits in the removal of suspended solids and microorganisms (Zhang et al. 2022)	The key factors influencing ultrafiltration performance include filtration cycle, flux, cleaning method, and the configuration of membrane modules. Among these, the packing density and effective length of membrane fibers hold the utmost importance in module design, serving as crucial areas of research for manufacturers (Zhang et al. 2022)
Constructed wetland	Substrate is saturated long mechanism that creates oxygen-poor conditions in the substrate, limiting the vegetation to those species that are adapted to low-oxygen environment (Oscar Omondi and Caren Navalia 2021)	Cost-effective and efficient wastewater treatment technology (Stefanakis 2018)	Among the primary drawbacks of wetland clogging is the decline in oxygen levels within the wetland, subsequently hindering the oxidation process and bacterial activity. This can lead to system failure and potentially reduce the designed lifespan by a considerable margin (Liu et al. 2016)
Oxic-anoxic-oxic	Strong nitrogen removal requires less oxygen for ammonium oxidation and less carbon source for nitrite reduction (Wang et al. 2016)	The combined anoxic and oxic process offers unmatched advantages, including stable operation and high efficiency, for the complete removal of nitrogen (Chen et al. 2023)	The substantial expenses associated with recirculation. The production of nitrogen oxides as end products, instead of N2, due to the microaerophilic conditions resulting from recirculation (Alzate Marin et al. 2019)

#### Wastewater treatment costs

The cost associated with wastewater management is a pressing concern, especially in light of the growing demand for this vital resource (Gallego Valero et al. 2018). Managing wastewater is not only costly but also raises questions about funding and the reduction of treatment expenses (Moral Pajares et al. 2019). Analyzing and studying the costs associated with different treatment methods is essential to enhance their efficiency, reduce costs, and facilitate their widespread adoption (San Juan et al. 2020). Typically, in wastewater treatment (WWT), the primary contributors to operational expenses include energy, chemicals, sludge disposal, and maintenance costs. Most case studies incorporate these cost elements when structuring their operational expenses. However, studies that integrate both environmental and economic assessments often do not provide detailed breakdowns of cost categories and may overlook replacement and end-of-life costs, making it challenging to conduct comparative analyses of cost categories at different stages of the life cycle. Moreover, cost categories vary among different WWT methods (Ilyas et al. 2021).

Rashidi et al. (2018) pointed out that economic analysis in the field of wastewater treatment plants (WWTPs) has received less attention compared to environmental or technological assessments, despite its significant role in the decision-making process (Rashidi et al. 2018). Identifying the optimal method is a complex task, as each treatment approach has its unique strengths and weaknesses. These aspects encompass not only cost considerations but also considerations related to effectiveness, practicality, environmental consequences, sludge generation, operational intricacies, prerequisites for pre-treatment, and the potential emergence of harmful by-products. Nevertheless, within the array of wastewater treatment methods, only a select few are widely embraced by the industrial sector, driven by both technological and economic justifications. From an economic perspective, chemical precipitation is both cost-effective and efficient among physicochemical methods. However, filtration often entails prohibitively high investment costs for small and medium-sized industries, and adsorption suffers from material costs and expensive regeneration. On the other hand, coagulation/flocculation offers the advantage of inexpensive capital costs (Crini and Lichtfouse 2019). In the realm of biological processes, the anaerobic–anoxic–oxic (AAO) process stands out for its low operating and management costs (Vo et al. 2022). Conversely, constructed wetland systems have established their credentials as environmentally sustainable and economically efficient technologies when compared to numerous other wastewater treatment approaches. These systems are engineered to mimic natural treatment processes within a controlled setting and hold a distinct advantage in terms of minimal to zero energy usage. A pivotal benefit of constructed wetlands is their elimination of energy requirements, which typically constitute the primary cost input in various wastewater treatment technologies (Waly et al. 2022).

## Discussion

### Physicochemical methods

**Fig. 2:**
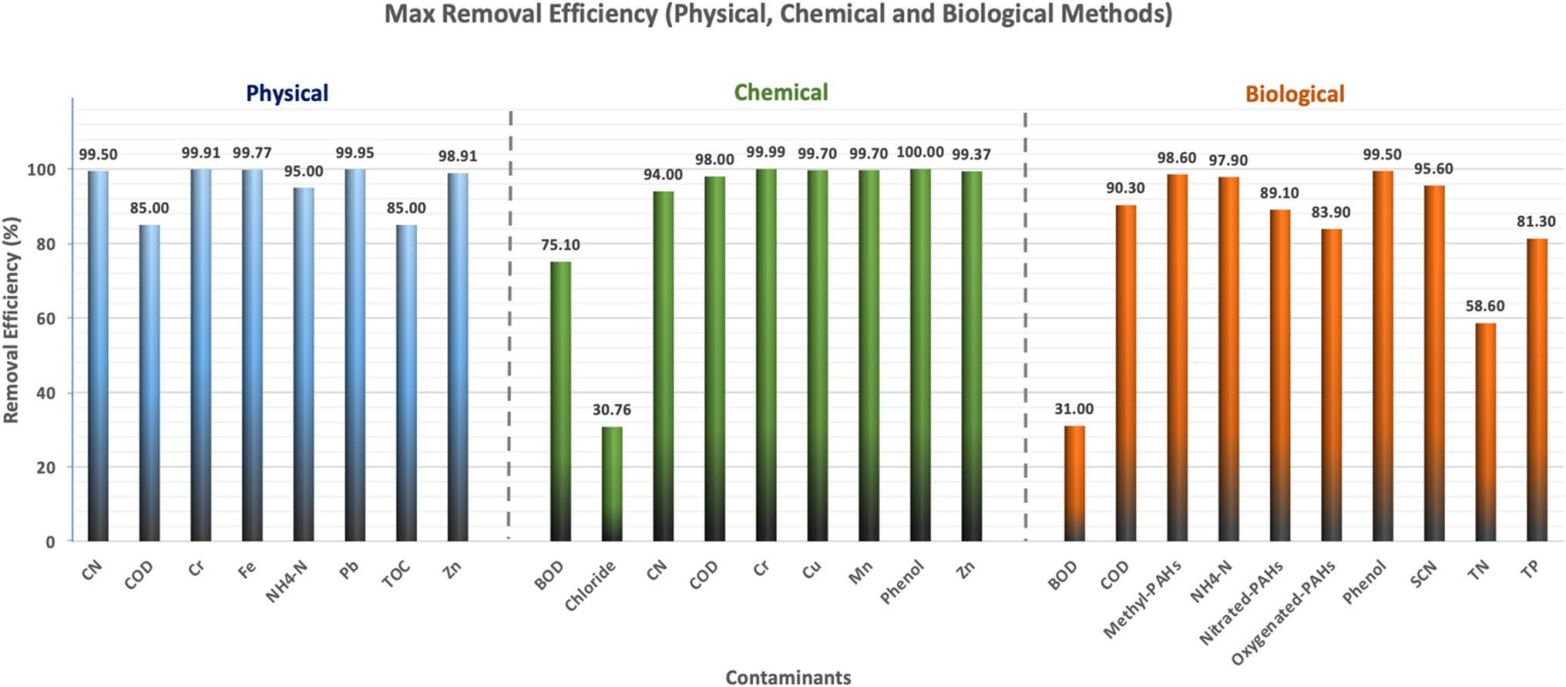
The effectiveness of biological processes in removing different contaminants

Researchers have consistently employed a range of physicochemical mechanisms, such as AOPs, coagulation, membrane processes, and precipitation, to effectively treat wastewater produced in various processes within the iron and steel industry. In Table 3, numerous physicochemical techniques investigated by researchers for treating effluents from the metallurgical industry are highlighted. Furthermore, Fig. 2 depicts the elimination efficiencies of different contaminants using physical, chemical, and biological techniques. Notably, physicochemical techniques demonstrate remarkable suitability for eliminating heavy metals with an efficiency exceeding 96%. Regarding biological processes, it can be observed that biological techniques achieve over 80% elimination rate for phenol and higher than 75% for COD, ammonium and cyanide.

**Table 3:** Physiochemical processes

Treatment method	Wastewater	Removal efficiency	Highlights	References
Physical method/adsorption	Steel industry	Fe^2+^: 99.7 Pb^2+^:99.9 Zn^2+^:98.9 Cr^6+^: 99.9	The treated clay exhibited consecutive reuse in four adsorption cycles while reducing the physicochemical variables to acceptable levels. Following the adsorption that, measurements indicated that the treated wastewater met the environmental requirements.	(Lawal Odebunmi and Adekola 2020)
Physical method/adsorption	Blast furnace	Cyanide: 99.5	The adsorption of cyanide on LDH exhibited a physisorption nature, with the dominating mechanism being ion exchange. The synthesized LDH material could be regenerated using a mixture of sodium hydroxide and sodium nitrate, allowing successful reuse for up to five cycles with an elimination rate of 85%. Additionally, the efficiency of LDH reuse could be enhanced by calcining it at 650°C.	(Ravuru et al. 2019)
Chemical method/electrocoagulation combined with photo-Fenton	Steel plant	COD:98 Phenol:100	The results clearly demonstrate the efficacy of the EC-PF process for eliminating organic substances such as phenol from water. The process exhibits superior elimination rate and proves to be more cost-effective when compared to alternative removal methods.	(Malakootian and Heidari 2018)
Chemical method/Photo-degradation and UV irradiation	Steel industry	Cyanide: 90	The presence of dissolved oxygen had a significant role in the degradation process. Initially, the rate of photo-decyanation was rapid, gradually decreasing over time. This observation ensures the process's industrial viability, as it indicates a sustainable and controlled degradation rate.	(Biswas et al. 2020)
Chemical method/ Ozonation	Coking	Cyanide: 94 COD: 72 BOD: 75	The integration of ozonation and EC proved highly efficient in lowering the levels of cyanide, COD and BOD. An initial cost analysis estimated a cost of 5.801 US$ per cubic meter (m^3^)	(Das, Anweshan, et al. 2021)
Physical method/filtration treated coking effluent	RO	COD: 85 ammonium nitrogen: 95 TOC: 85	The phytotoxicity was notably lower after treatment via a sand bed and reverse osmosis (RO). After just the sand bed, the wastewater still exhibited toxicity. Among the treatment methods, the reverse osmosis process resulted in the lowest toxicity levels observed in the wastewater.	(Smol et al. 2018)
Chemical method/chemical precipitation	Stainless steel	Cu:99.7 Mn: 99.7	The research demonstrated that the electric arc furnace dust slag by itself is capable of significantly removing the target metals, thereby lowering the expenses associated with acquiring expensive chemicals. The addition of a small quantity of Ca(OH)_2_ resulted in the elimination of both metals, achieving an impressive efficiency of 99.7%.	(Forsido et al. 2020)
Chemical method/precipitation	Plating	Cu: 93.9 Zn: 99.3 Cr^3+^: 99.9	Sulfide precipitation exhibits superior elimination capabilities for mixed heavy metals, resulting in lower levels of total suspended solids when compared to hydroxide precipitation.	(Yatim et al. 2021)

Smol et al. aimed to evaluate the effectiveness of integrated membrane processes for removing specific pollutants from coke wastewater and to assess the impact of coke wastewater on the germination of the test plant, broad bean (Vicia faba), on two substrates: cotton and Murashige and Skoog basal medium (MSBM). The results confirmed the efficient treatment of coke wastewater in the presented system, which included sand bed filtration and reverse osmosis. A clear correlation was observed between increasing concentrations of wastewater and seed germination of the test plant. Notably, significant removal efficiencies were achieved for key parameters: sand bed filtration reduced COD by 39%, TN by 46%, TOC by 42%, TC by 47%, SS by 50%, and 16PAHs by 53%. After reverse osmosis, COD was reduced by 85%, TN by 95%, TOC by 85%, TC by 85%, SS by 98%, and 16PAHs by 67%. However, it is crucial to consider not only basic physical and chemical indicators but also their impact on test organisms. Coke wastewater collected from the biological treatment plant exhibited high levels of germination inhibition (90–98% for the cotton matrix and 92–100% for the MSBM matrix), indicating strong toxicity with a toxicity rating of 3. Even after treatment on a sand bed, the wastewater remained toxic, with germination inhibition ranging from 24 to 48% for the cotton matrix and 14–54% for the MSBM matrix, and a toxicity rating of 2. The toxicity of wastewater was lowest after reverse osmosis treatment, with germination inhibition in the range of 4–10% for the cotton matrix and 2–12% for the MSBM matrix, and a toxicity rating not exceeding 1. Biologically treated wastewater exhibited significantly higher phytotoxic effects compared to wastewater treated with a sand bed and reverse osmosis (Smol et al. 2018).

Mining operations are substantial contributors to pollution due to the discharge of saline water containing heavy metals, high sulfate levels, hardness, ammonia, oil, and elevated TOC concentrations. The primary method for managing these discharges involves the use of reverse osmosis (RO) to purify the water, ensuring it complies with discharge regulations before being released into other water bodies. However, handling RO concentrates poses a significant challenge (Y. Li et al. 2017; Pervov et al. 2023).

To address the issue of RO concentrate, a zero liquid discharge (ZLD) process has been introduced. This process involves chemically softening the RO concentrate and further evaporating it. However, the ZLD approach incurs high energy costs and faces challenges due to the large volumes of water that need treatment. Moreover, mining industries are often located in remote northern regions, resulting in substantial expenses for reagent supply. Pervov et al. have embarked on the task of developing an innovative approach aimed at significantly decreasing the flow of reverse osmosis (RO) concentrate. This reduction is achieved through the incorporation of a series of nanofiltration membranes, ultimately resulting in a total dissolved solids (TDS) value of 110–120 g/l. The obtained concentrate is then mixed with wet sludge, which is subsequently dewatered and removed in conjunction with the dehydrated sludge. Experiments have revealed a reduction in the concentration of calcium in the concentrate, attributed to the precipitation of calcium carbonate on “seed crystals” during the circulation process. Another distinctive aspect of this novel technique is the division of the concentrate into two separate streams, one containing high concentrations of monovalent ions (sodium and ammonium chlorides) and the other comprising divalent ions (calcium, magnesium, and copper sulfates) (Pervov et al. 2023).

### Adsorption

Adsorption is primarily a surface phenomenon that encompasses three main processes: (a) film diffusion, which involves the movement of adsorbate molecules from the fluid phase to the external surface of the adsorbent, (b) pore diffusion, which entails the migration of adsorbate molecules from the external surface into the pores of the adsorbent, and (c) the accumulation of the adsorbate and subsequent physical/chemical interaction with the active locations present on both the outer surface and within the pores of the adsorbent (Srivastava et al. 2021).

**Fig. 3:**
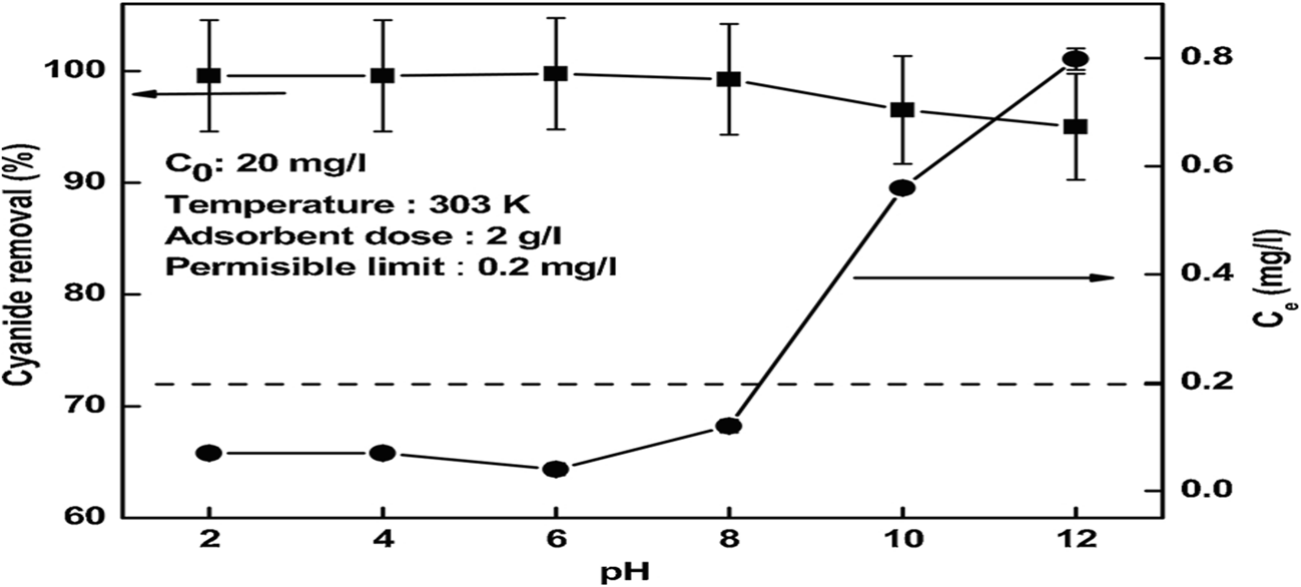
The impact of solution pH on the elimination of cyanide by LDH. Reprinted with permission from (Ravuru et al. 2019)

Ravuru et al. conducted a study on the synthesis of nickel aluminum intercalated with nitrate for the purpose of adsorbing cyanide from the effluent. The experimental findings revealed that the maximum adsorption reached 166 mg/g. Additionally, the impact of solution pH was explored, and the outcomes are depicted in Fig. 3. It was observed that the cyanide elimination rate remained consistent (99.6%) within the pH range of 2–6. However, an additional rise in pH resulted in a 4% decrease in cyanide elimination (Ravuru et al. 2019).

Upon examining the findings, it becomes evident that adsorption is a well-researched and effective method for removing various contaminants, such as Cr, *CN*^−^, Mn, F, and others, from effluents produced by the steel industry. Additionally, the utilization of side-products created at various stages of steel manufacturing to create cost-effective adsorbents can address the problem of disposing the sludge. By placing more emphasis on adsorbent regeneration, conducting small scale studies, and modifying adsorbents to target particular contaminants, adsorption can be widely implemented for the treatment of steel industry effluent (Ravuru et al. 2019).

In a study conducted in 2019, the adsorption ability of WS-EAF slag was assessed for the elimination of cadmium and manganese from wastewater samples from a WWTP treating industrial wastewater. The experiments conducted at optimal parameters resulted in impressive removal efficiencies of 99.6% for manganese and 99.9% for cadmium. Furthermore, the study revealed the potential of WS-EAF for removing other heavy metals (zinc, iron, nickel, lead, and carbon monoxide) from wastewater (Abd El-Azim et al. 2019).

Lawal Odebunmi and Adekola (2020) conducted a study where they created a cost-effective adsorbent using K-S clay modified with NaOH to target the elimination of metal ions from wastewater originating from the steel industry. The experimental results demonstrated impressive elimination rates, reaching 99.77% for Fe^2+^, 99.95% for Pb^2+^, 98.91% for Zn^2+^, and 99.91% for Cr^6+^ (Lawal Odebunmi and Adekola 2020).

Pattanaik et al. conducted a separate study where they experimentally treated coke wastewater from a steel plant, which contained a high concentration of phenol, utilizing Mobil Composition of Matter no.41. Their findings revealed that the use of this nanomaterial proved efficient in removing 96% of phenol, accompanied by a 90% reduction in COD. However, further examination and scrutiny are required to evaluate the suitability of implementing such a model for industrial purposes (Pattanaik et al. 2018).

### Electrocoagulation

Electrocoagulation (EC) is a highly efficient electrochemical method employed for treating wastewater originating from various sources. During this process, the application of a difference in voltage disrupts the pollutant charge. Additionally, metal ions are released, that serve as coagulants, leading to the creation of flocs. These flocs can be subsequently eliminated through precipitation or flotation methods (Tahreen et al. 2020).

According to Syam Babu et al. (2020), wastewater treatment through electrocoagulation exhibits a 10 to 15% higher efficacy compared to conventional chemical coagulation. Similarly, Oden and Sari-Erkan (2018) conducted comparable studies on the treatment of wastewater from the metal plating industry. They reported significant elimination rates reaching almost 99.9% for dyes and zinc and 96% for nickel (Oden and Sari-Erkan 2018).

Malakootian and Heidari conducted a study to assess the feasibility of eliminating phenol through a combined electrocoagulation (EC) and precipitation filtration (PF) process. The experimental investigation, demonstrated remarkable removal efficiencies of 100% for COD and 99% for phenol within the system. In contrast to previous studies, a reduction in elimination rate was observed as the CD increased, which could be attributed to electrode polarization and reduced electrode activity. Additionally, an analysis of energy requirements during the process highlighted the importance of optimizing the CD value for effective pollutant removal (Malakootian and Heidari 2018).

### Advanced oxidation processes

Advanced oxidation processes (AOPs) are extensively employed for the treatment of both organic and inorganic contaminants present in wastewater. These processes utilize various methods such as catalysts, irradiation, and ultrasounds to activate oxidants, generating highly reactive free radicals with strong oxidization capabilities. These free radicals effectively degrade or mineralize the organic molecules found in the wastewater, contributing to the overall purification process (Song et al. 2022).

Wang et al studied the treatment of coking wastewater that was subjected to EC, using electrodes doped with varying amounts of Yb (up to 5 mol%). The optimal Yb quantity was determined to be 1.5 mol%, resulting in an electrode with a smooth surface and increased crack formation, thereby enhancing the specific surface area (SSA) and active sites. Under other optimal conditions, the elimination rates for COD and TOC were found to be approximately 85% and 60%, respectively. In order to improve the electrocatalytic capability, there is a growing interest in the use of novel, cost-effective electrode materials. Consequently, electrooxidation offers promising opportunities for efficiently treating organic materials and toxic substances found in wastewater from the steel industry (Wang et al. 2022).

Photodegradation has emerged as a widely employed technique in wastewater treatment, owing to its numerous advantages. These advantages include robust radical-based reactions, rapid elimination rates, full degradation of pollutants and ability to recycle (Majumder et al. 2021). Biswas et al. conducted a study on the photocatalytic degradation of cyanide using H_2_O_2_ and UV irradiation. H_2_O_2_ contributes to the photodegradation of CN_−_ as react together to form OCN^−^, and finally be converted to bicarbonate and nitrogen. The results indicated a high rate of photodegradation during the initial 30 min, which gradually decreased over time. By the time the 3-h test was finished, the concentration of *CN*^−^ was below 1 mg/L, demonstrating an elimination rate of over 95%. Interestingly, when H_2_O_2_ and UV irradiation were separately employed, they proved to be incapable of removing *CN*^−^ (Biswas et al. 2020).

The Fenton process has demonstrated its effectiveness in degrading organic compounds found in wastewater (Silva and Baltrusaitis 2021). In another study by Teixeira et al., steel wool in the form of zero-valent iron (ZVI) was introduced as a possible catalyst for oxidizing phenol via H_2_O_2_. Under optimal conditions and a 2-h reaction period, the tests revealed that the phenol concentration decreased to lower than 0.5 mg/L, achieving an elimination rate of 99.75%. Additionally, it was noted that increasing the temperature by 20 degrees, resulted in a reduction of the reaction time from 2 to 1 h. However, further increases in H_2_O_2_ concentration did not lead to an improvement in phenol removal efficiency (Teixeira et al. 2015).

**Fig. 4:**
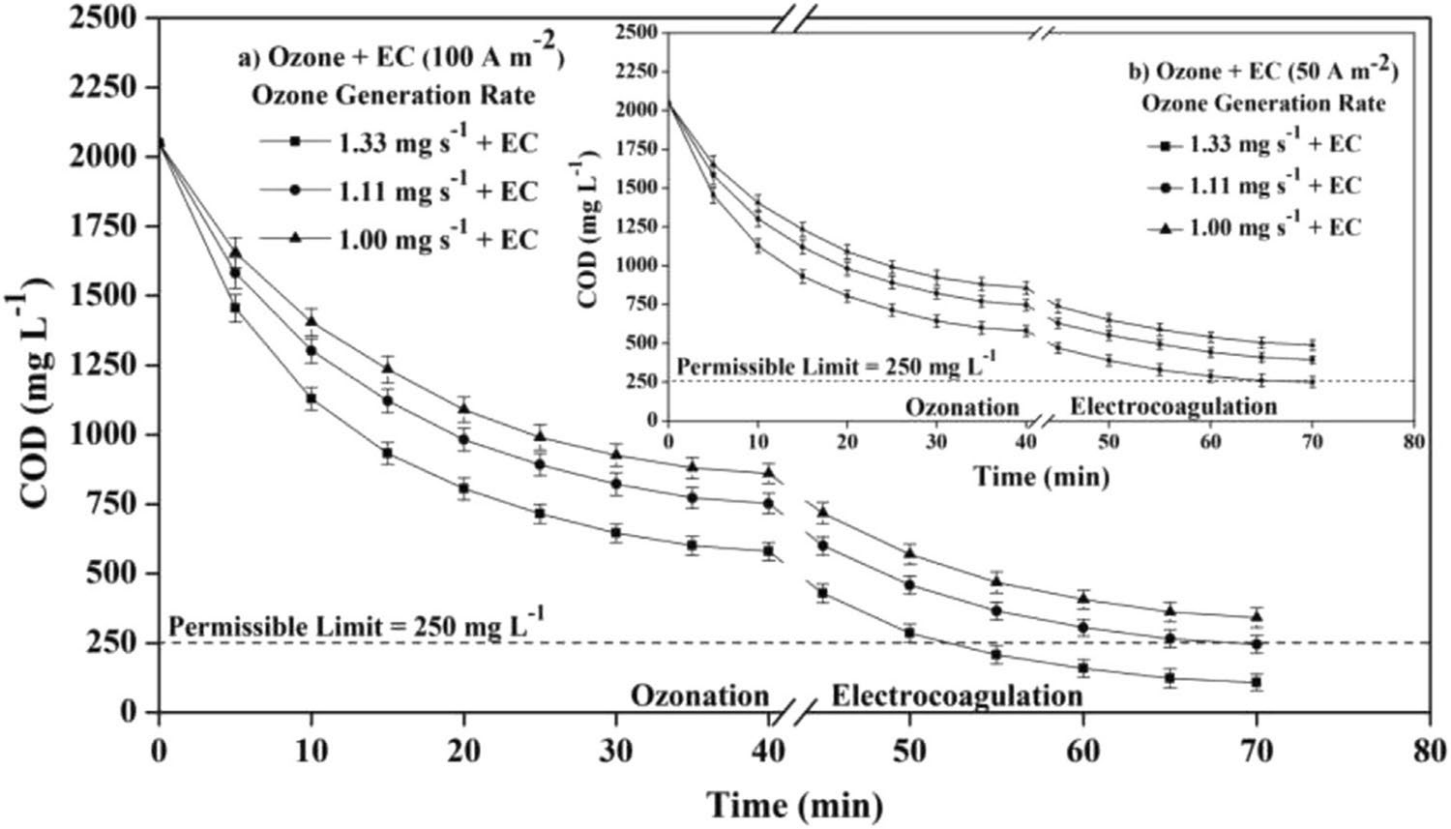
The impact of ozone generation rates on COD elimination over time was examined in the hybrid process, with a current density of 100 Am^−2^. The inset graph displays the results obtained at a CD of 50 Am^−2^. Reprinted with permission from (Das et al. 2021a)

Ozonation is a treatment process that is influenced by pH, wherein molecules of ozone or free radicals interact with specific pollutants to reach the desired results (Cunha et al. 2022). An example of this is seen in the research conducted by Das et al., where a ozonation/EC combination was employed to treat the effluent produced by a steel plant. Figure 4 illustrates the efficiency of COD elimination as a function of ozone production rate over time. During the EC process, the COD quantity decreased to 110 mg/L (Das et al. 2021a).

The findings suggested that the combination of processes lead to higher elimination rates compared to the conventional electrocoagulation (EC) method. Specifically, removal efficiencies of 99.8% for *CN*^−^, 94.7% for COD, and 95% for BOD were obtained. Furthermore, it is notable that the ozone production rate and current density were two significant factors that exhibited a direct relationship with cyanide elimination. Increasing the ozone generation rate from by 0.33 mg/s resulted in a 17.3% improvement in removal efficiency. However, it was noted that the decline in *CN*^−^concentration ceased after 40 min, with no further significant *CN*^−^elimination observed. Integrating EC and ozonation is a more cost-effective and efficient approach compared to using ozonation alone. Limited mineralization of refractory organics, low ozone yield from generators, and the inherent instability of ozone, which poses risks during storage and transportation are the major challenges related to ozonation (Das et al. 2021a).

### Membrane processes

Recent advancements have led to significant improvements in membrane wastewater treatment methods (Mao et al. 2021), which have garnered considerable attention as a post-treatment technique to further refine treated wastewater (Smol et al. 2018).

Pervov proposes the application of the recently developed technique for mitigating hardness in RO concentrate through the utilization of NF processes operated in a recirculation mode. To address this challenge, the authors employed four previously established techniques, as elaborated and documented in their prior work, which enable the reduction and “concealment” of RO concentrate (Pervov et al. 2023).

- Limiting the concentrate volume reduction to a narrow range, not surpassing 0.4–0.5% of the original feed water volume, and then removing the concentrate alongside the dewatered sludge.
- Reducing the concentrate volume and inducing the precipitation of calcium carbonate with the aid of “Seed Crystals”.
- Reducing the concentrate volume by employing a sequence of low-rejection membranes.
- Dividing the concentrate into two separate solutions, specifically concentrated solutions of monovalent and divalent salts, to facilitate their subsequent use.

**Fig. 5:**
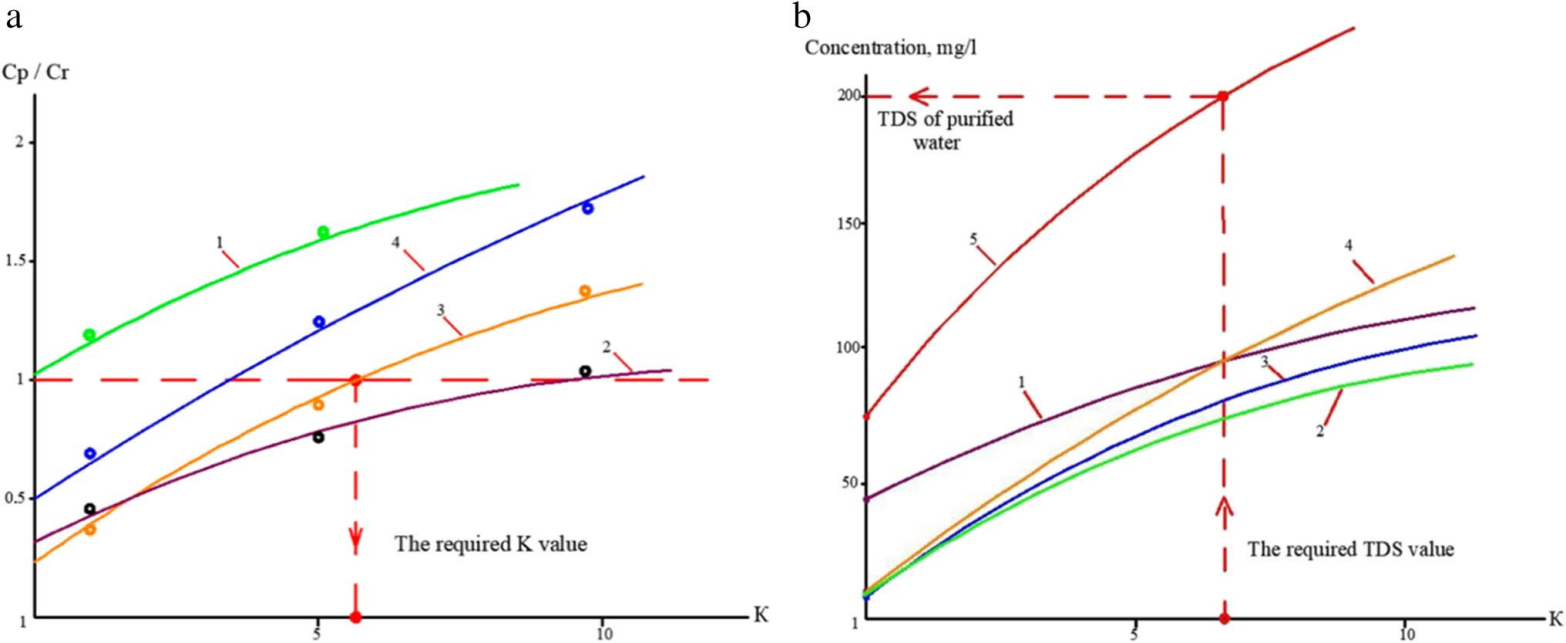
**a** The correlation between Cp/Cr ratio values and K values for various membranes utilized in the initial stage of membrane treatment: 1—ammonium ions in RO permeate; 2—ammonium ions in NF permeate; 3—copper ions in RO permeate; 4—copper ions in NF permeate. **b** The interrelation of concentrations of different constituents in the product water of the reverse osmosis membrane with *K* values in the first phase of membrane treatment and the assessment of the necessary recovery value: 1—chloride; 2—sodium; 3—calcium; 4—sulfate; 5—total dissolved solids (TDS) (Pervov et al. 2023)

The results of various species assessments in the concentrates and permeates, contingent on *K* values, are illustrated in Fig. 5a and b. The figure illustrates the relationships between specific concentration ratio values for ammonia and copper. To determine the second-stage recovery, we identified the *K* value corresponding to the point where the copper concentration surpasses the regulatory threshold in Fig. 5 (a). With this chosen K value, we can calculate the permeate’s total dissolved solids (TDS) and the concentrations of various other substances, as depicted in Fig. 5 (b). Figures (a) and (b) display the values of different ions and impurity concentrations that offer insights into predicting the chemical composition.

The deposition of calcium carbonate onto seed crystals serves to decrease TDS and facilitates further concentration enhancement. Experimental results demonstrate the attainment of high TDS levels, with a reduction in concentrate volume by less than 1% of the initial volume. This portion can be allocated to both sludge dewatering facilities for the dewatering of suspended sludge and the precipitation of calcium carbonate (Pervov et al. 2023).

In their study, Smol et al. examined the effectiveness of a combination of sand bed filtration and reverse osmosis (RO) for removing residual contaminants. The findings demonstrated that passing the pre-treated effluent through the RO system resulted in the elimination of 85% of COD and 67% of PAHs. Additionally, the RO effluent exhibited the lowest level of toxicity. The researchers emphasized the importance of tracking physicochemical indicators in wastewater samples and their effects on the organisms present (Smol et al. 2018).

Yang et al. conducted a study on the elimination of thallium ions using the emulsion liquid membrane (ELM) method. The system consisted of 2-ethylhexyl phosphoric acid-2-ethylhexyl ester (P507) as the carrier and aviation kerosene as the solvent. Several influential variables, such as the concentration and extraction time were investigated and optimized for their impact on the elimination rate of thallium. The maximum removal capacity of thallium, reaching 99.7%, was achieved within a quarter of an hour under optimal conditions (Yang et al. 2017).

Wang et al. utilized a dual membrane method, combining ultrafiltration and nanofiltration, to effectively remove targeted pollutants. Prior to going through the membranes, the treated wastewater underwent sand filtration. The experimental findings indicated that the concentrations of COD and ${NH}_4^{+}-N$ in the effluent were both below 60 and 2 mg/L, respectively (Wang et al. 2017). Thanks to the advancement of nanofiltration membranes, there is now an opportunity to concentrate discharged brines and effluents with the aim of further utilization, as demonstrated by Havukainen et al. in 2022. Pervov and his research team conducted a comprehensive review of prior studies conducted within their department, which revolved around the application of reverse osmosis and nanofiltration processes. These processes extended beyond just wastewater purification, encompassing activities such as the treatment of silt water, sewage sludge dewatering, and the filtration of leachates from solid waste landfills. They also involved the management of membrane plant concentrates and the removal of all substances captured by the membranes, in addition to the handling of dehydrated sludge.

By employing nanofiltration membranes with low rejection characteristics, they achieved remarkable results in terms of high recovery rates and elevated total dissolved solids (TDS) levels in the concentrate. The deposition of calcium carbonate on seed crystals played a crucial role in reducing TDS and facilitating further concentration augmentation. Experimental outcomes demonstrated the ability to attain high TDS levels while simultaneously reducing the concentrate volume by less than 1% of the initial volume. This remaining fraction could be allocated to both sludge dewatering facilities for the dewatering of suspended sludge and the precipitation of calcium carbonate. Pervov and their co-authors showcased the results of their comprehensive experimental program, which aimed to minimize the volumes of all liquid waste generated during mine water treatment. Their innovative approach involved utilizing a cascade of nanofiltration membranes, ultimately achieving a TDS value ranging from 110 to 120 g/l (Pervov et al. 2023).

### Coagulation and precipitation

The precipitation/coagulation process is commonly employed for the elimination of suspended solids, organic materials, COD, oil and color. This process consists of two phases: firstly adding a coagulant in order to neutralize the charge of colloidal particles, and secondly, formulating larger flocs that ultimately precipitate out of the solution (Alazaiza et al. 2022).

Yatim et al. conducted a comparative study to assess the efficacy of hydroxide and sulfide precipitation methods for removing metals (copper, zinc and chromium) from wastewater generated in the plating industry. The results of the study demonstrated that sulfide precipitation achieved a 7.3% higher removal efficiency for Cu compared to hydroxide precipitation. However, no notable change was observed for the elimination of zinc and chromium. It is noteworthy that the sulfide precipitation process resulted in a higher total suspended solids (TSS) compared to the hydroxide precipitation method (Yatim et al. 2021).

Xue et al. implemented a bioinspired method known as bioinspired calcium carbonate precipitation (MICP) to encapsulate heavy metal ions. This approach involved the introduction of ureolytic bacteria, yeast extracts, and calcium sources. Remarkably, the researchers reached a 100% remediation efficiency for Pb^2+^ and Cu^2+^ ions when operating under ideal conditions (Xue et al. 2022).

In a study conducted in 2020, EAF dust slag was employed as a substitute for typical alkalis for the neutralization acidic effluent generated by a steel processing plant. The findings showed that the target metal (copper and manganese) concentration was reduced by more than 84% and 90% respectively. The pH was raised to 8.7, effectively neutralizing the effluent. Furthermore, at higher pH levels, copper formed insoluble precipitates while soluble manganese was converted into insoluble manganese substances (Forsido et al. 2020).

**Fig. 6:**
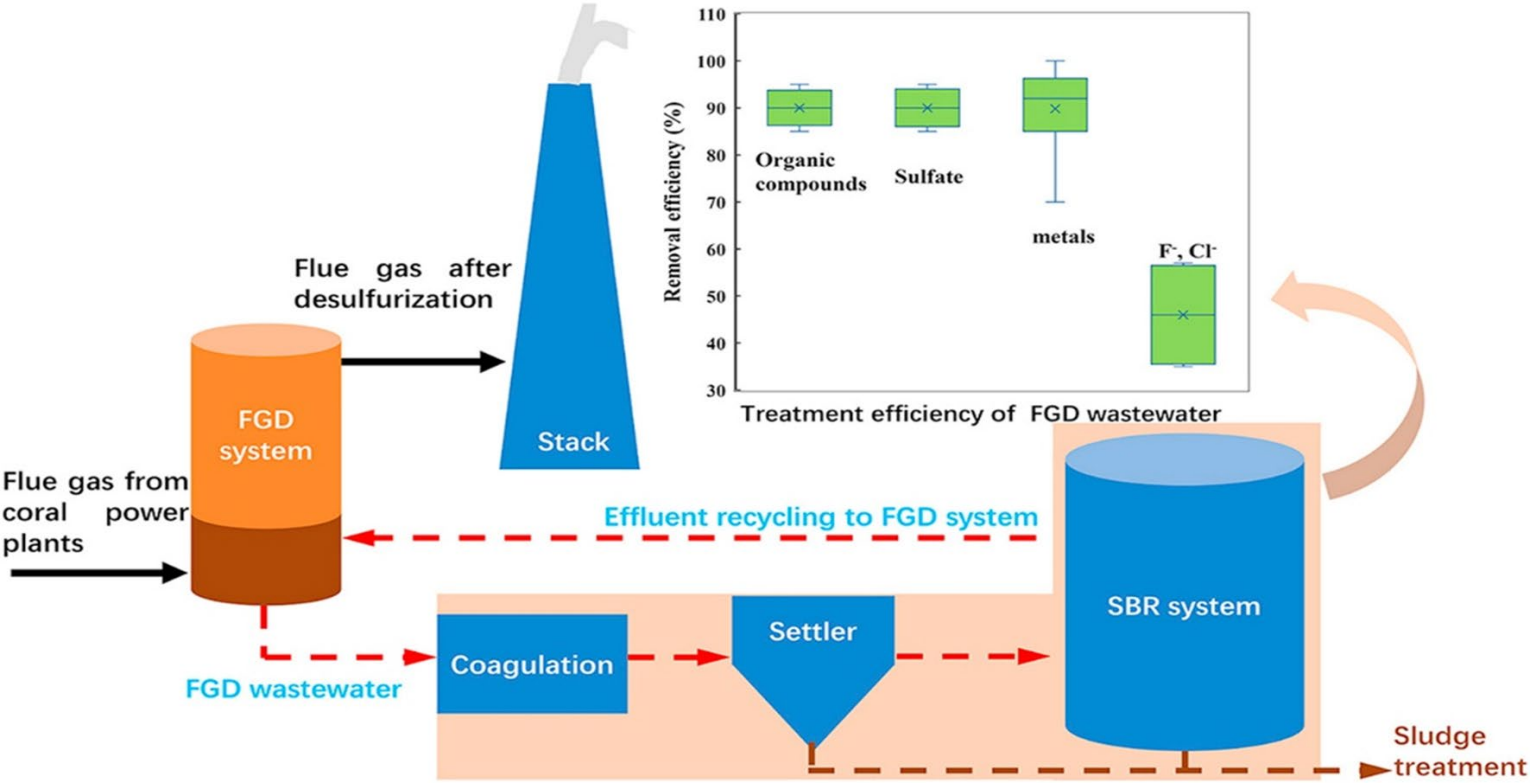
Flue gas desulfurization process in coal-fired power plants. Reprinted with permission from (Yan et al. 2019)

According to the study of Yan et al. (2019), a comprehensive chemical precipitation and sulfate reduction (CP-SR) process was established to treat effluents from flue gas desulfurization (FGD) systems in coal-fired power plants, achieving 97% suspended solids removal. FGD process in coal-fired power plants, produces wastewater with concentrated sulfate, chloride, metals, and refractory organic compounds. Simultaneously, removal of chlorine and fluoride was achieved, possibly associated to co-precipitation or biosorption processes. The procedure followed for the treatment of this type of waste is shown in Fig. 6 (Yan et al. 2019).

Excessive Cl− concentration can impede limestone dissolution and impact gypsum quality, resulting in a reduction in desulfurization efficiency [48]. Consequently, the Cl− concentration is typically maintained within the range of 12,000–20,000 ppm in desulfurization systems. FGD wastewater characterized by low pH levels contains elevated chloride levels, substantial sulfate content, suspended solids, hardness ions, heavy metals, and other constituents, presenting a significant challenge in terms of treatment (X. Liu et al. 2023).

Improper discharge or inadequate treatment of wastewater with high chloride concentrations can give rise to a series of ecological and environmental issues, with potential repercussions for human health. (a) Hypersaline wastewater can hinder the growth of agricultural crops and plant seeds, leading to soil salinization (Cao et al. 2022), (b) The direct discharge of chloride-containing wastewater into water bodies can result in increased water mineralization (Song et al. 2020a, 2020b), (c) Due to the inhibitory effects of high Cl− concentrations on microorganisms, hypersaline wastewater is typically treated using physico-chemical methods (Figler et al. 2019).

Chloride removal from FGD wastewater has consistently posed challenges (Liu et al. 2023). While chemical precipitation through the addition of copper slag or silver salt, as well as electrochemical methods, are effective means of chloride removal in FGD wastewater, their industrial applicability is limited due to economic considerations (Polo et al. 2020). Membrane separation techniques (including concentration-driven, pressure-driven, and electrical-driven membrane methods) have the capacity to efficiently eliminate Cl−. However, these technologies have faced criticism regarding their high initial investment, maintenance costs, and energy consumption (Li et al. 2022). Ion exchange systems are susceptible to interference from other ions, resulting in reduced Cl− removal efficiency (Paul and Chang 2020). Solvent extraction represents a straightforward and effective separation method, albeit with relatively higher costs (Zhang et al. 2020a, 2020b). Oxidation approaches for Cl− treatment do not lead to secondary contamination, and the oxidation products can be repurposed for disinfectants. Nevertheless, these methods are not without drawbacks, including low utilization rates of oxidants, high operational expenses, and elevated chloride concentrations in the treated water (Zhang et al. 2020a, 2020b). Currently, precipitation methods are the most widely adopted and efficient means of removing Cl− from hypersaline wastewater among the available techniques, owing to their high efficiency and ease of operation.

### Biological treatment technologies

The substantial volumes of wastewater produced across various sections of the steel industries necessitate the implementation of cost-effective and efficient treatment. Biological methods rely on the activity of microorganisms for the degradation of organic compounds and removal of nutrients found in the wastewater. This process can take place under aerobic or anaerobic conditions. The techniques can be further categorized into suspended growth processes, such as the activated sludge process (ASP), which suspends the biomass, or attached growth processes, which utilize a biofilm for the treatment of industrial wastewater (Srivastava et al. 2021). Various methods utilized for the biological treatment of metallurgical effluents are summarized in Table 4.

**Table 4:** Biological processes for wastewater treatment

Treatment technology	Type of wastewaters	Removal efficiency	Highlights	References
Biological fluidized bed reactor	Coking	Ammonium nitrogen: 90, Phenol: 99, COD: 85	To assess the stability of nitritation under varying organic loading rates (OLR), a long-term experiment was conducted over a period of 200 days using a biological fluidized bed reactor (BFBR). The experimental data aligned with the predictions of a mathematical model, which identified the optimal conditions for achieving stable nitritation.	(Li et al. 2021)
Constructed wetland	Stabilizing pond effluent	BOD_5_:31 TN 15+ Ammonium nitrogen 90+	The effluent from the constructed wetlands exhibited decreased measurements of all measured parameters. The highest elimination rate was observed for ammonium nitrogen (>90%).	(Nguyen et al. 2019)
Oxic-anoxic-oxic, combined with chemical oxidation and sedimentation	Coking	MPAHs: 98.6 OPAHs: 83.9 NPAHs: 89.1%	For most low molecular weight methylated PAHs, PAHs, as well as some oxygenated PAHs and nitro PAHs, the main elimination mechanism was biodegradation. On the other hand, adsorption by dewatered sludge played a significant role in removing high molecular weight PAHs, as well as many OPAHs and NPAHs. However, to further eliminate the remaining semi-volatile PAHs (SPAHs) and PAHs in the treated effluents, advanced treatment methods are necessary.	(Saber et al. 2021)
Constructed wetland	Treated steel rolling	COD: 61 TP: 81 TN: 58	The results indicated that the horizontal flow constructed wetland (HFC) demonstrated effective elimination of turbidity, COD and total phosphorus. However, the average elimination rate of total nitrogen was only about 25%. It was observed that increasing the hydraulic retention time led to varying degrees of improvement in the elimination rate of contaminants in both HFC and horizontal flow subsurface flow artificial wetland (HFSAD).	(Zheng et al. 2021)
Anoxic/oxic/anoxic/oxic	Coking	COD: 90 and 87, Ammonium nitrogen 97 and 88	High molecular weight PAHs were found to be the predominant compounds in the untreated coking wastewater. These compounds exhibited significant degradation in the sequencing batch reactor with anoxic/oxic/anoxic/oxic configuration compared to the typically used anoxic/oxic/oxic process. The superior performance of the former in treating total nitrogen can be attributed to the higher abundance of Thiobacillus, SM1A02, and Thauera, which are likely the key factors driving the enhanced TN elimination in this system.	(Fan et al. 2021)

### Constructed wetlands and bioremediation

Constructed wetlands have gained significant popularity as a cost-effective and efficient wastewater treatment technology over the years (Stefanakis 2018). Nguyen et al. studied the ability of constructed wetlands (CWs) in improving the quality of effluent used for industrial wastewater treatment. The findings indicated an elimination rate of 90% for ${NH}_4^{+}-N$, 31% for BOD, 28% for COD, and 15% for TN. Furthermore, the constructed wetland system achieved greater than 60% elimination of Mn and 45% elimination of Fe (Nguyen et al. 2019).

**Fig. 7:**
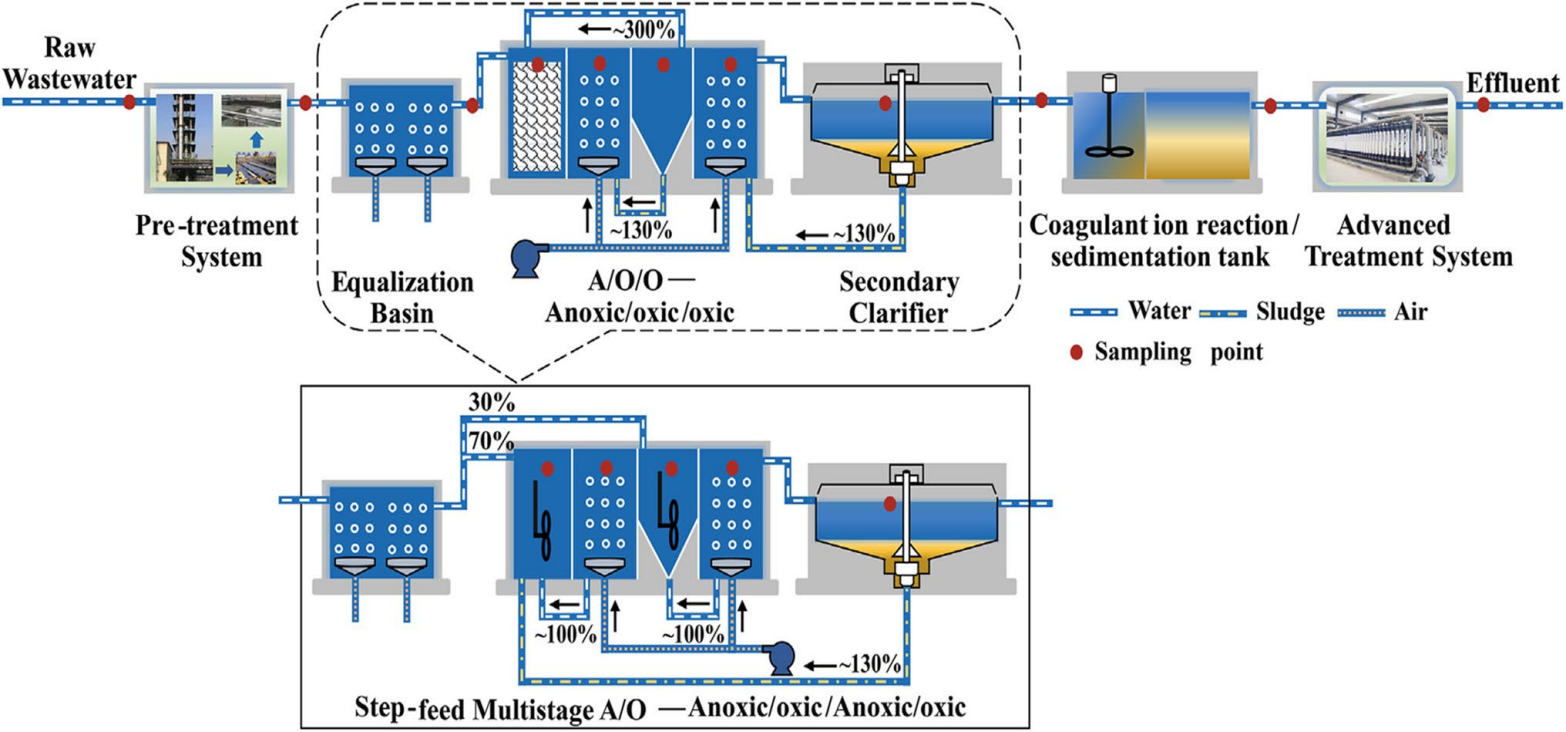
Schematic depiction of the configuration of the treatment systems for coke wastewater. Each system consists of three distinct treatment components. Reprinted with permission from (Fan et al. 2021)

After undergoing pre-treatment for decyanation and oil elimination, the untreated coke wastewater was introduced into the biological systems. In order to ensure the availability of organic carbon for denitrification in A2, a step-feeding strategy was employed, with 70% of the influent directed to A1 and the remaining 30% to A2. Reactor A in the A/O/O process was specifically created for the biofilm process, with a polyethylene filler occupying approximately 65.0% of its volume. On the other hand, both A1 and A2 in the SF-A/O/A/O process were created as activated sludge processes and contained stirring devices. Following the reconstruction, the A/O/O process and the SF-A/O/A/O process were operated simultaneously for comparative purposes (Fan et al. 2021). This is illustrated in Fig. 7.

In order to enhance the denitrification ability of constructed wetlands for treating steel rolling wastewater, Zheng et al. implemented a combination of submerged aerobic denitrification with horizontal subsurface flow constructed wetlands. The first section of the system contained manganese sand and gravel (HFC), while the other of comprised ceramic, sulfur, and lime. The findings demonstrated notable removal efficiencies, with overall percentages of 93.5% for turbidity, 61.4% for COD, 81.3% for TP, and 58.6% for TN. Importantly, the inclusion of the second section within the constructed wetland system contributed to a significant 20% improvement in TN elimination (Zheng et al. 2021).

In an investigation by Rai et al., the effectiveness of phycoremediation was examined as a third-tier method for the synchronous elimination of phenol, cyanide, and ammonia nitrogen from coke-oven effluent that had undergone secondary treatment. The researchers utilized a microalgal strain (Tetraspora sp. NITD 18) to treat simulated wastewater. The findings demonstrated that under optimal conditions, the reported removal efficiencies were approximate 79% for phenol, 74.7% for ammonia nitrogen, and 80.4% for cyanide. Notably, when real wastewater was diluted, the elimination rate improved by approximately 27.5% (Rai et al. 2021).

**Fig. 8:**
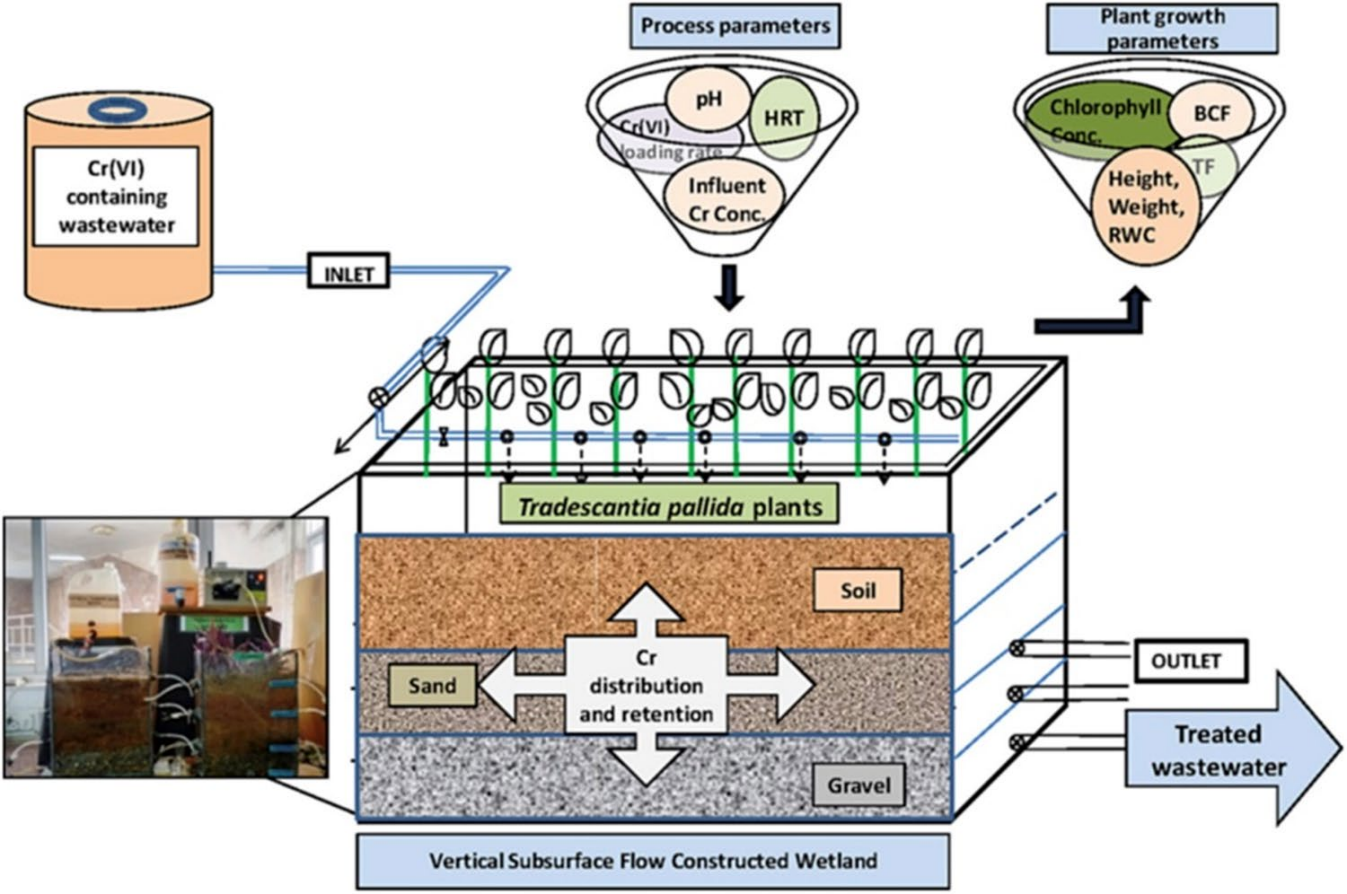
Schematic of the vertical flow constructed wetland experimental setup for Cr(VI) removal. Reprinted with permission from Sinha et al. (2017)

In addition, huge amounts of tannery industry wastewater, containing a high concentration of chromium (Cr), and constructed wetlands (CWs) are considered as an environmentally friendly technique to treat this wastewater (Younas et al. 2022). Furthermore, the study of Sinha et al. (2017) showed that wastewater containing Cr(VI) can be treated using constructed wetlands (*T. pallida* plants as subsurface) under continuous operation mode and the relative schematic of the set up used and is illustrated in Fig. 8. According to the results, *T. pallida* plant biomass–based CW system showed a high percentage of Cr (VI) removal (97.2%) in comparison to control CW.

Hexavalent chromium (Cr(VI)) is recognized as one of the most harmful metals contaminating soil and water resources. Compounds containing chromium are released into the environment primarily through anthropogenic sources due to their wide-ranging commercial applications. These compounds find extensive use in industries such as leather tanning, metallurgical operations, and steel production. Leveraging the *T. pallida* based constructed wetland system, which offers both high Cr removal efficiency and cost-effectiveness in terms of construction and operation, presents an effective solution for treating wastewater containing chromium from tanneries, metallurgical facilities, textile plants, and various other industrial sectors (Sinha et al. 2017).

### Combined biological techniques

Li et al. introduced a new strategy for achieving stable nitritation within a fluidized bed reactor. They accomplished this by harnessing the toxic substances found in coking wastewater as inhibitors of nitrite-oxidizing bacteria. The study observed that the performance of ammonia-oxidizing bacteria was more sensitive to phenol concentrations under varying organic loading rates, while high concentrations of thiocyanate adversely affected the performance of nitrite-oxidizing bacteria. Additionally, the elimination rates of COD, phenol, and thiocyanate were tracked, resulting in elimination rates of 85.8%, 99.5%, and 95.6%, respectively (Li et al. 2021).

In their study, Saber et al. investigated the presence and elimination of PAHs in wastewater treatment plants that employed anoxic and oxic zones to treat coking wastewater. The research revealed high elimination rates, ranging between approximately 89 and 98%. This elimination was primarily attributed to the adsorption process followed by the biodegradation occurring under anoxic conditions (Saber et al. 2021).

## Metallurgical slag for wastewater treatment

**Fig. 9:**
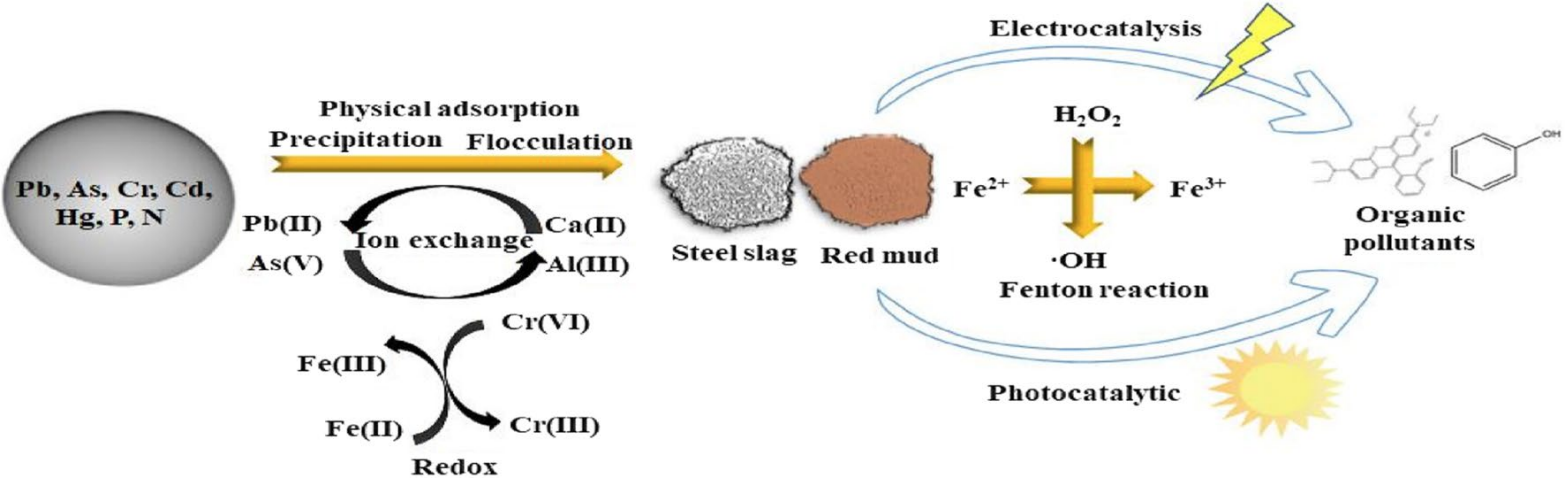
Depiction of the mechanism with which steel slag and red mud materials remove pollutants. Reprinted with permission from (Ji et al. 2022)

The utilization of metallurgical slag for the elimination of contaminants in wastewater is regarded as an effective approach to implement the concept of “using waste to treat waste”. Metallurgical slag possesses significant storage capacity and is economically viable. However, it also poses environmental risks and threatens human safety due to the leaching of heavy metals from the slag (Ji et al. 2022). Figure 9 illustrates the mechanism diagram depicting the elimination of pollutants by steel slag and red mud materials.

The considerable storage capacity and potential for secondary utilization have made blast furnace slag (BFS) an area of significant interest as a side product of the iron and steel production process (Ji et al. 2022). Mohammed et al. conducted a study in which blast furnace slag (BFS) was modified with sulfamic acid to produce zwitterionic ion functionalized BFS (Z-BFS). The elimination ability of Z-BFS was tested for chromium ions and methylene blue dye. The results indicated that Z-BFS exhibited favorable removal performance for chromium ions and methylene blue dye at pH values greater than 5. Under optimized conditions, the extraction efficiencies exceeded 90% for both contaminants. After three regeneration cycles, the removal efficiency of chromium ions decreased to 65%, and that of methylene blue dye decreased to 80% (Mohammed et al. 2021).

Copper slag (CS) is produced during copper smelting (Ji et al. 2022). Wu et al. conducted a study where they synthesized a core-shell composite called CS@polyaniline (CS@PANI). The resulting material had a magnetization value of 6.6 emu/g, making it easily to separate from the liquid phase. The researchers investigated the adsorption performance of the composite for chromium. The highest theoretical adsorption capacity of chromium at 323.15 K was calculated to be approximately 452 mg/g, which was consistent with the actual results of about 445 mg/L. After 5 cycles of chromium elimination, the efficiency only dropped from approximately 98% to about 90% (Wu et al. 2021).

Electrolytic manganese slag (EMS), a side product generated during the electrolytic production of metal manganese, contains ammonia nitrogen and heavy metals. If not properly treated, direct exposure of EMS can pose a significant threat to the surrounding environment (Ji et al. 2022). Li et al. conducted a study where they modified EMS to create a catalyst called MS-N_3_H. This catalyst was designed to improve the degradation effectiveness of LVF. It is porous and has a large specific surface area. The maximum adsorption capacity of MS-N_3_H for LVF was determined to be approximately 16 mg/g, resulting in a removal rate of 23%. Upon attaining adsorption equilibrium, the introduction of peroxymonosulfate initiated the catalytic reaction, resulting in the degradation of approximately 59% of LVF and achieving an overall removal rate of approximately 82%. Even after undergoing four cycles, the efficiency of LVF removal remained steady at 77%. To assess the effectiveness of LVF elimination using MS-N_3_H, its efficiency was tested in various types of water, resulting in removal efficiencies of ranging between 72.6 and 77.7%. An analysis revealed that manganese exhibited the highest activity among the elements on the surface of MS-N_3_H (Li et al. 2020).

## Economic considerations

According to Das et al., the operating cost of the integrated EC and ozonation processes, which accounted for energy requirements and electrode cost for EC, was estimated to be 5.801 US$/m^3^. This cost was found to be comparable to other hybrid processes reported in the literature. Considering that the steel manufacturing industry is highly dependent on water, it is important to explore opportunities for reducing fresh water consumption (Das et al. 2021a). Zhang et al. created models for integration of water networks in the steel industry. They were successfully applied, resulting in a decrease of 22% in freshwater consumption and a 20% reduction in the total network. In order to treat NF reject water, the inclusion of the RO technique with precipitation incurred a cost of approximately 7.4 $/m^3^ (Zhang et al. 2018). The implementation of integrated techniques, such as UV-H_2_O_2_, for the elimination of *CN*^−^ was found to reach a cost of approximately 4.6 $/m^3^ of water per hour. While these options have shown effectiveness, further research is needed to accurately estimate the overall implementation cost, taking into account various factors that can influence costs (Deepti and Purkait 2021).

From an environmental standpoint, metallurgical slag is considered a form of solid waste. Disposing of it through landfilling or stacking not only causes pollution but also requires expensive land occupation. In China, solid waste production enterprises without dedicated storage facilities are charged $4 per ton of waste. The cost of treating and managing red mud accounts for 2% of alumina production costs. Transforming metallurgical slag into a valuable resource can effectively address waste treatment using waste, which holds great promise from both environmental and economic perspectives. Currently, the cost of frequently used commercial adsorbents, such as almond, walnut and coconut shells, rice husk, and corncob, is significantly higher than that of metallurgical slag. The production cost per ton for these materials ranges between $1540 and $3900 (Ji et al. 2022).

## Conclusions

The steel industry produces a significant volume of wastewater, necessitating the careful consideration of suitable treatment technologies, factoring in the source, composition, contaminant concentration, and discharge standards. This review focuses on the technologies and processes used for metallurgical waste treatment. Key pollutants found in the wastewater include heavy metals and xanthates. Therefore, it is crucial to effectively extract these substances from the wastewater before it is reused or discharged.

This review comprehensively introduces and discusses the various technologies and processes employed for the treatment of organic waste present in water from metallurgical processes. We emphasize key factors that play a crucial role in determining the effectiveness of integrated systems. This information will be invaluable for industrial practitioners seeking to establish a sustainable treatment process capable of addressing a mixture of contaminants originating at different stages within the steel manufacturing process. Furthermore, this review underscores the importance of not solely aiming to meet discharge standards but also striving to achieve water of reusable quality when dealing with effluent treatment in the steel industry. Membrane technologies, in this context, emerge as a viable solution, and the selection of these technologies should consider factors such as the intended use of water, energy consumption, and cost implications.

In recent years, the implementation of more stringent regulatory standards has garnered increased attention from both academia and industry. This has led to a growing interest in advanced technologies, including highly efficient oxidation, reverse osmosis (RO), and nanofiltration (NF). However, it is worth noting that RO treatment introduces the challenge of concentrate disposal. Addressing the issue of concentrate disposal and exploring potential utilization avenues remains a pending challenge in cases where concentrate discharge is not feasible. In certain situations, existing techniques designed to reduce or eliminate concentrate prove to be prohibitively expensive, particularly when factors like limited access to power and chemicals come into play. A novel concept involves the idea of “hiding” concentrate within the dewatered sludge. This approach effectively eliminates liquid waste by separating mine water into a purified water stream that can be safely discharged into natural water bodies and a dewatered sludge that is directed to landfill. Additionally, this technological scheme eliminates the need for chemical softening and concentrate evaporation, resulting in significant operational cost savings. Moving forward, future research endeavors should prioritize the development, modification, regeneration, and sustainable management of spent adsorbents in order to address the evolving challenges in wastewater treatment.

Chemical precipitation and adsorption methods are commonly employed for the extraction of heavy metal ions from wastewater, which is particularly effective versus chromium, copper, mercury, nickel, zinc, and other heavy metals. Among the various treatment processes currently cited for wastewater treatment, only a few are commonly employed by the industrial sector for technological and economic reasons. Defining a one-size-fits-all method for removing all pollutants from wastewater is challenging. Currently, no single method can effectively treat all contaminants, primarily because industrial effluents are inherently complex. In practice, a combination of various methods is frequently employed to achieve the desired water quality in the most cost-effective manner.

### Supplementary information

ESM 1 (PDF 122 kb)

ESM 2 (PDF 2759 kb)

ESM 3 (PDF 122 kb)

ESM 4 (PDF 122 kb)

ESM 5 (PDF 123 kb)

ESM 6 (PDF 123 kb)

## Abbreviations

ASP: Activated sludge process
AOPs: Advanced oxidation processes
BOD: Biological oxygen demand
BFBR: Biological fluidized bed reactor
BICP: Bioinspired calcium carbonate precipitation
COD: Chemical oxygen demand
CP-SR: Chemical precipitation and sulfate reduction
CS: Copper slag
EAF: Electric arc furnace
EC: Electrocoagulation
EC-PF: Electro coagulation–photo Fenton
EMS: Electrolytic manganese slag
ELM: Emulsion liquid membrane
FGD: Flue gas desulfurization
HFC: Horizontal flow constructed wetland
HFSAD: Horizontal flow subsurface flow artificial wetland
K-S clay: Kaolin clay
LDH: Layered double hydroxide
LVF: Levofloxacin
NPAHs: Nitrated-PAHs
OLR: Organic loading rates
OPAHs: Oxygenated-PAHs
PAHs: Polycyclic aromatic hydrocarbons
PF: Precipitation filtration
RO: Reverse osmosis
SSA: Specific surface area
SPAHs: Substituted polycyclic aromatichydrocarbons
TN: Total nitrogen
TSS: Total suspended solids
WS-EAF: Water-spray electric arc furnace
Yb: Ytterbium
ZVI: Zero-valent iron
Z-BFS: Zwitterionic ion functionalized BFS
