# Supplementary material for: Advancements and sustainable strategies for the treatment and management of wastewaters from metallurgical industries: an overview
Source: Environ Sci Pollut Res Int. 2023 Nov 14;30(57):119627–53. doi: 10.1007/s11356-023-30891-0 (PMC10697902; doi:10.1007/s11356-023-30891-0)
Supplement: Supplementary file 2 — (PDF 2759 kb) [file 11356_2023_30891_MOESM2_ESM.pdf]

membranes

Review

Recovery of Metals from Wastewater—State-of-the-Art Solutions with the Support of Membrane Technology

Katarzyna Staszak and Karolina Wieszczycka *

Institute of Chemical Technology and Engineering, Faculty of Chemical Technology, Poznan University of Technology, ul. Berdychowo 4, 60-965 Poznan, Poland
* Correspondence: karolina.wieszczycka@put.poznan.pl

Abstract: This paper discusses the most important research trends in the recovery of metals from industrial wastewater using membrane techniques in recent years. Particular attention is paid to the preparation of new membranes with the required filtration and separation properties. At the same time, possible future applications are highlighted. The aspects discussed are divided into metals in order to clearly and comprehensibly list the most optimal solutions depending on the composition of the wastewater and the possibility of recovering valuable components (metalloids, heavy metals, and platinum group metals). It is shown that it is possible to effectively remove metals from industrial wastewater by appropriate membrane preparation (up to ~100%), including the incorporation of functional groups, nanoparticles on the membrane surface. However, it is also worth noting the development of hybrid techniques, in which membrane techniques are one of the elements of an effective purification procedure.

Keywords: membrane filtration; metals rejection; metals recovery; platinum group metal; heavy metals; wastewater treatment

check for updates

Citation: Staszak, K.; Wieszczycka, K. Recovery of Metals from Wastewater—State-of-the-Art Solutions with the Support of Membrane Technology. *Membranes* **2023**, *13*, 114. https://doi.org/10.3390/membranes13010114

Academic Editors: Xanel Vecino and Mònica Reig

Received: 30 December 2022
Revised: 13 January 2023
Accepted: 13 January 2023
Published: 16 January 2023

Copyright: © 2023 by the authors. Licensee MDPI, Basel, Switzerland. This article is an open access article distributed under the terms and conditions of the Creative Commons Attribution (CC BY) license (https://creativecommons.org/licenses/by/4.0/).

1. Introduction

Membrane techniques are now well established in industrial applications, especially in the food industry [1,2]. Factors that favour this are, above all, their low-emission characteristics. This is why the use of membranes is recommended in the Best Available Technologies (BAT) manual for wastewater treatment [3]. The development of membrane techniques and, consequently, the increasing use of membranes is due to the development of new membrane materials that are adapted to specific processes requirements [4]. The fact that membrane techniques remain not only within the sphere of application of researchers is confirmed by an increasing, but still low, number of literature reports indicating research also using real industrial wastes on a pilot scale. One direction of this research is the use of membranes for the removal and recovery of metals from aqueous solutions. This is of particular importance because, as is well known, wastewater that contains metal ions is hazardous and requires very precise and efficient treatment methods [5,6].

Figure 1 shows an example of an industrial effluent with possible metals present. A detailed analysis of this wastewater in terms of treatment options is discussed later in this paper. However, this illustration already shows how wide a spectrum of metals can be present in sewage. Furthermore, it is to be expected that these metals are often present together in solution, which significantly limits the possibility of a selective separation of individual compounds.

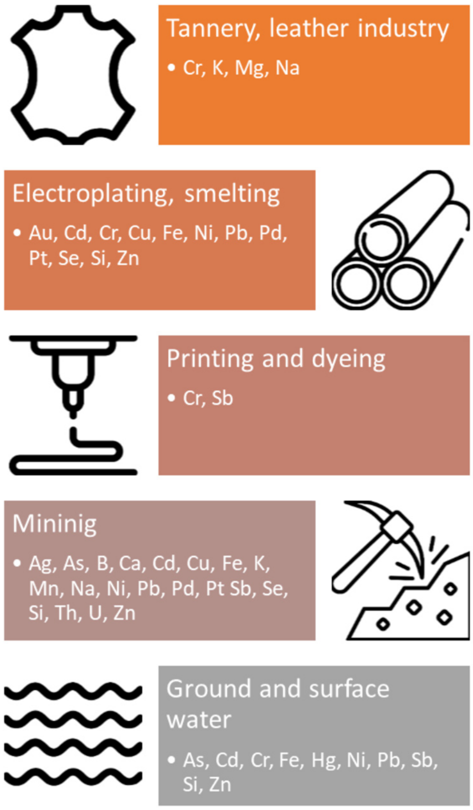

Figure 1. Occurrence of metals in industrial wastewater and secondary ground and surface water.

The paper focuses on recent reports that address the role of membrane techniques in the treatment of industrial wastewater. The selected methods, which are described in detail in the following paper, are schematically illustrated in Figure 2. It should be noted at this point that an analysis of the literature indicates several shortcomings in this area. Most studies are conducted on model solutions. This approach results from a number of factors. First, it must be agreed with the researchers that model tests give us a relatively quick and simple answer as to whether the proposed separation method is adequate. For this reason, most of the research works in the development stage focuses on model tests. In addition, research works are also conducted in an attempt to reproduce the composition of actual industrial effluents. These studies, which are already more complex, definitely bring the possibility of evaluating a process for its potential application in industrial practice closer. On the other hand, the same scientists do not always have access to industrial wastewater. Although efforts are made to tighten the cooperation between science and industry, this is not always possible. Here, it is important to bear in mind issues of corporate secrecy, their know-how, or the limitations of scientific units to work on a larger scale. Therefore, all those works that try to describe and analyse real industrial processes or investigations using laboratory-scale equipment based on real wastewater should be appreciated in spite of these difficulties.

The dominance of pressure-driven membrane processes in wastewater treatment (see Figure 2), such as reverse osmosis (RO), nanofiltration (NF), and ultrafiltration (UF), should not come as a surprise, as these are the processes that are most commonly used in industrial applications [7]. This is mainly due to the fact that these processes are mature and, consequently, suitable membranes or complete solutions can be easily obtained [8]. At the same time, it is increasingly observed that membrane techniques support other available separation techniques. Hybrid systems that combine membrane processes with other conventional separation technologies, for example, precipitation, as presented in Figure 2, offer excellent opportunities to reduce energy consumption and minimise environmental impact.

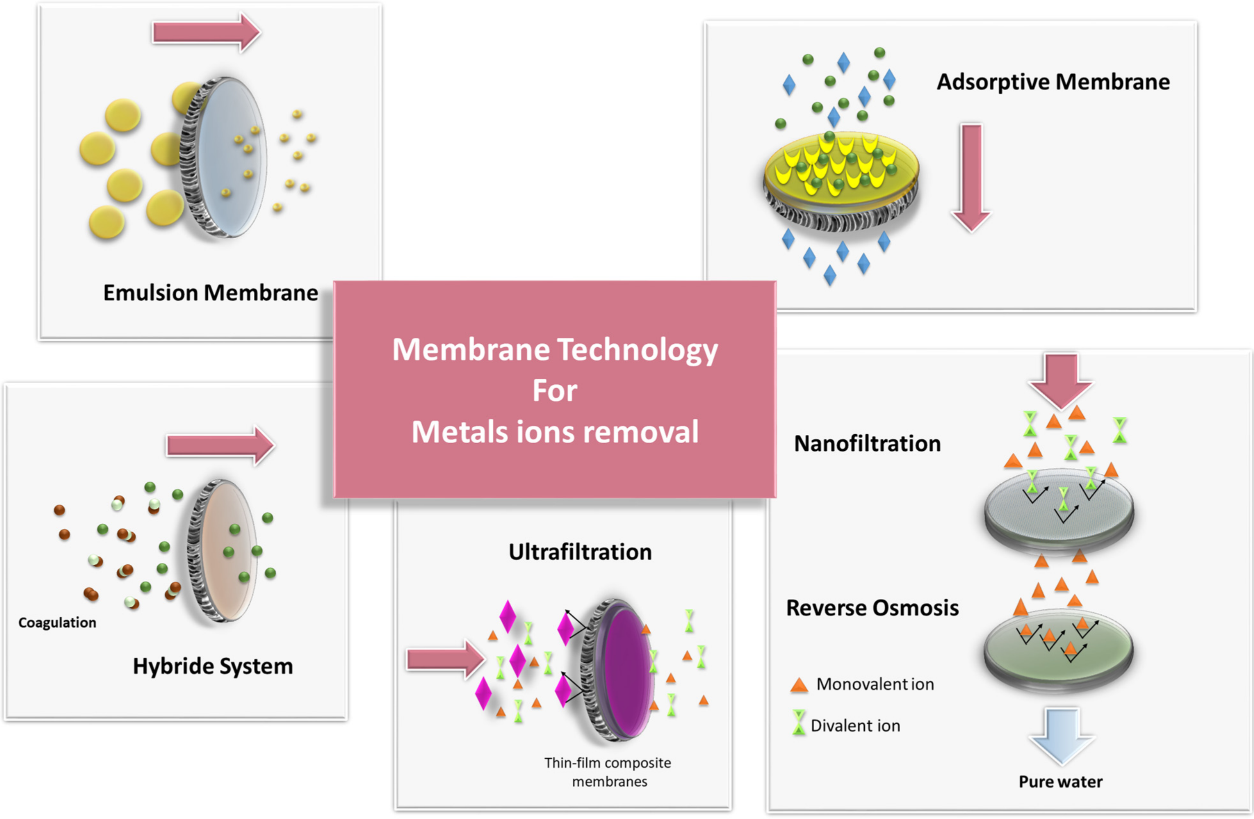

Figure 2. Membrane-based methods for metal removal from industrial wastewater.

The intention of this review was to create a compendium of knowledge on the possibilities of using membrane techniques for wastewater treatment containing metals. Figure 3 shows all 25 elements discussed in this review.

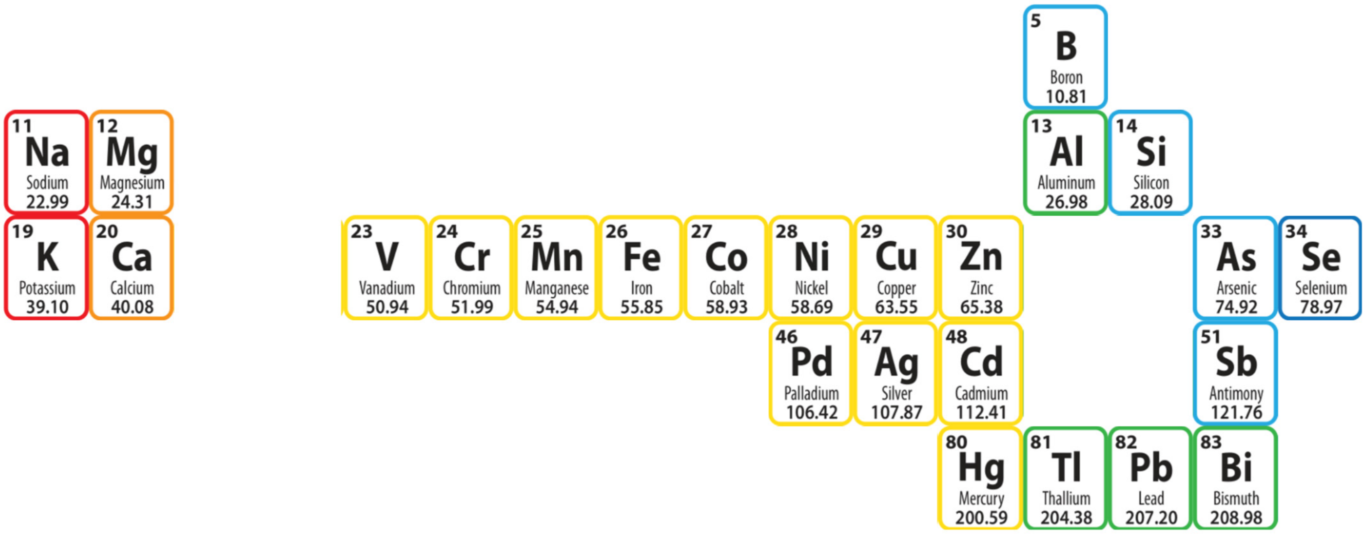

Figure 3. Metals considered in the paper.

2. Removal of Metalloids

2.1. Arsenic (As)

Arsenic is a metalloid that exhibits an extreme toxic potential with serious health consequences [9]. Exposure to arsenic occurs not only through groundwater or sewage, but in recent years the permissible concentrations of arsenic have also been exceeded in

natural water systems such as rivers and lakes. The choice of techniques also depends on the speciation of arsenic, among others, the high efficiency against As(V) is due to the fact that As(III) is predominantly non-charged at pH levels below 9.2 (As(III) mostly exists as H_3AsO_3, while the primary arsenate species are monovalent $H2AsO_4^-$ and divalent $HAsO_4^{-2}$) [10]. The relatively low concentration of arsenic impurities and the effectiveness of their reduction make membrane technologies the most advantageous of all those recommended [11]. In addition, recent studies have identified nanofiltration (NF), reverse osmosis (RO), and membrane distillation (MD) as the most promising technologies for the production of high quality drinking water and wastewater treatment [12,13]. In recent years, attention has been paid not only to optimizing the parameters of the filtration process using commercial membranes (NF-1, NF-45 [14], NF-90 [15], NF-300 [16], NF90-4040 [17], UTC-70, NTR-7450 [18]), but also to testing the impact of various types of their modifications, especially on increasing the selectivity and efficiency toward arsenates [19]. In the case of the commercially NF, most of the available membranes have a constant surface charge, which results from the presence of dissociating groups, e.g., carboxylate and sulfonate. This enables ion separation through a combination of various effects (pore size, ion interaction mechanisms, and electrical effect). The porous polyamide thin-film nanocomposite membrane NF90-4040 (Dow Filmtec) is one of the few commercial membranes that is characterized by a high rejection efficiency of both arsenate and arsenite species (reduction from 50–300 μg/L to a limit value of 10 μg/L As) [17]. The tests have also shown that the efficiency is significantly dependent on the operating temperature (the removal of As(III) and As(V) decreased as the temperature increased) and the pressure (removal increased with an increase in pressure). Both parameters probably affect the diffusivity of arsenic. As already indicated above, membrane performance parameters far from expectations, especially in terms of arsenite removal, were the motivator for the development and application of new membrane fabrication techniques and their modifications. The most interesting results obtained for the NF-PS-3 membrane are a thin-film polyamide coated microporous polysulfone [20]. The modification carried out ensured the higher removal of both arsenates and arsenites from an aqueous solution containing concentrations of As than those recommended for commercial membranes. Finally, a rejection of As(V) and 70.4% of As(III) was achieved for aqueous feed containing 1 mg/L of As. Further modifications, especially the incorporation of nanoparticles into the structure of the polyamide membrane, directed the filtration materials to remove As(III). Moreover, these studies concern not only arenates, but also selenium and boron compounds, which are characterized by similar chemistry and difficulty in removal. Removal of selenium from the aquatic environment is a particularly complex and costly process due to the complexity of the sewage (mainly post-mining) and the presence of selenium compounds at various oxidation states (Se(IV) and Se(VI)) [21]. An example of novelty in filtration materials is a membrane produced as a result of intercalation of chitosan-clay nanoparticles (C-SBF) in the PA/PEG structure [22]. The PA-CSBF4 membrane showed improved pure water permeability and rejections for As(lll) and selenium ions (99 and 98%, respectively, at a permeate flux of 444 L/m^2 h) due to the increasing hydrophilicity of C-SBF nanoparticles. In another work, He et al. studied the influence of the incorporation of sodium ion modified carbon quantum dot (Na-CQD) on NF polyamide membrane to reject Se and As ionic species [23]. The fabricated filtration material had both high pure water permeability (10.4 LMH/bar) and rejection of SeO_3^{2-}, SeO_4^{2-} and $HAsO_4^{2-}$ at 98, 98 and above 99%, respectively. He et al. also employed UiO-66 (Zr-MOF) as nanofillers on the polyamide thin-film nanocomposite's membrane to remove SeO_3^{2-}, SeO_4^{2-} and $HAsO_4^{2-}$ with a flux of 11.5 LMH/bar and with the ions rejection higher than 96% [24]. In another work, the researchers explored the water-soluble zwitterionic copolymer of 2-methacryloyloxyethyl phosphorylcholine and 2-aminoethyl methacrylate hydrochloride incorporated into the polyamide selective layer of thin-film composite membranes. The thin-film composite NF membranes obtained exhibited lower pure water permeability than that observed for PA-CSBF4 (8.5 LMH/bar), but the rejection of SeO_3^{2-}, SeO_4^{2-} and $HAsO_4^{2-}$ was compa-

rably high (98.2, 99.1 and 99.8%, respectively) [25]. Ultrafiltration (UF) membranes have been shown to have pores too large to reject arsenate. The solution was to embed UiO-66 particles in the membrane matrix. This increased the adsorption capacity of the membrane and allowed the removal of arsenate during membrane filtration [26]. Much worse results in the rejection of As(V) obtained using polyphenylsulfone hollow fiber membranes with cellulose acetate or cellulose acetate phthalate as low-cost ultrafiltration additives. The percentages of arsenic removal depended on the additive used, and were 34% and 41% with permeabilities of 44.42 L/m^2h bar and 40.11 L/m^2h bar, respectively [27]. Due to the complexity of waste solutions and the low efficiency as well as selectivity of ultrafiltration, a hybrid approach was proposed as a support, the use of a readily available, inexpensive adsorbent. Removal of As(V) by co-precipitation with Fe(III) oxides/hydroxides, followed by low-pressure membrane filtration, is a solution to reduce As to a concentration below 1 μg/L [28]. This process is characterized by high efficiency in the removal of As(V) (even at high phosphate and silicate concentrations), and this is due to both the fact that the Fe(III) particles and coprecipitation products are much larger than the membrane pore size, and that the As(V) co-precipitation reaches equilibrium before membrane filtration. Unfortunately, the disadvantage of this solution is the formation of an Fe(III) cake layer on the membrane surface, which reduced the permeability of the membrane [28]. The interaction with Fe was also used to remove As(III), although in this case the ceramic membrane with incorporated iron (montmorillonite-pearlite-iron ceramic membrane) was investigated [29]. The results show that the addition of iron improves the removal of As(III), but a much higher content reduces the adsorption capacity as a result of the reduction of porosity and permeability, competition of phosphates and carbonates, and changes in the polarization of the membrane surface. The detailed results for the removal of As, as well as other metalloids, from wastewater are shown in Table 1.

2.2. Boron (B)

Boron is the most common element in ocean salts, with concentrations ranging from 0.5 to 10 mg/L. Like most metalloids, boron is found in groundwater, mainly as a result of leaching from rocks and soils. On the other hand, anthropogenic sources of water-soluble boron are agrochemicals (e.g., pesticides, fertilizers) and detergents [30]. Due to its wide occurrence in ocean waters, until recently, dissolved boron was effectively removed by thermal desalination. This method allowed the removal of almost all the boron content, but due to its energy consumption, the method is now gradually being given up [31]. In nature, boron occurs mainly in the form of boric acid, borates, or borosilicate. Boric acid is the most soluble form, which gains anionic form only at a strongly alkaline pH. This property is quite a significant problem in boron removal operations. It is also significant that the physicochemistry of boric acid makes membrane processes ineffective at pH below 9, or requires at least two membrane steps or hybrid systems, while a high pH value can favor scaling phenomena [31]. Boron removal from seawater can be achieved at neutral pH by employing a multipass RO process. Kürklü et al. [32] proposed a multistage process for concurrent simultaneous desalination and boron removal (CDBR) to overcome the problems of requiring chemicals, low overall process recovery, and high energy demand in RO technologies. Operating tests confirmed that for seawater containing 10 ppm of boron and 35.000 ppm of other salts, the CDBR process (equipped with commercial RO or NF membranes) at OPD 74.5 bar can achieve a reduction in boron to a level of 0.5 ppm and salts to 100 ppm, and an overall water recovery of 70–75% [33].

In the case of membrane modification, the use of novel hydroxyl-terminated poly (ethyleneimine) in place of a commercial polyphenylene sulfone membrane increased the rejection of boron of the UF process from 6.4 to 45% [34]. In another work, Kumar et al. [35] used the phosphonic acid derivative of titanium dioxide as an inorganic filler of the polyamide layer on an ultrafiltration polyethersulphone support. The modification provided 2.5 times greater boron rejection than the commercial NF90 membrane and a high potential to reject scalant ions. The removal of boron and arsenic from saline geothermal

water was also conducted using a novel adsorption–hollow-fiber UF membrane hybrid system, in which N-methyl-D-glucamine functionalized resin was used as an adsorbent [36]. This system allowed the removal of boron in 86%, although it depended significantly on the amount of resin used and indicates the complexity of the process, which reduces the application potential of the solution. In addition, the use of adsorption polymers requires additional separation or regeneration.

2.3. Silica (Si)

The silica content in industrial water is a serious problem because of its deposition in pipelines, heat exchangers, or filtration elements. In the case of desalination membranes, silica deposits lead to a deterioration of membrane performance. This is a significant reason to extend the drinking water treatment process with a unit that allows the reduction of silica concentration to 150 mg/L, ensuring the solubility of silicates. One of the solutions tested is a hybrid continuous stirred tank reactor (CSTR) adsorption/ultrafiltration system using iron oxy/hydroxide as agglomerates and a hollow fiber UF membrane served as a barrier to the passage of the adsorbent, which has enabled the removal of silica from brackish groundwater in a relatively short residence time (15 min) [37]. The tests indicated that adsorption increased with increasing silica concentration from 25 to 70 mg/L and decreased with increasing concentration of agglomerates (limit 2.5 g/L). Moreover, with a constant stream of water, the UF membrane was not fouled in the presence of nanosized agglomerate particles. The disadvantage of this process is, however, that the concentration polarization decreases during the early stages of cross-flow filtration. Colloidal silica can also be removed by ultrafiltration using a membrane with a molecular weight cut-off range of approximately 10,000 Da. An example is the removal of silica from surface water using the HFS 60 Silica module ensuring a removal rate of 99.8% at a throughput of two streams 6000 m^3/day [38]. An identical module was installed in the Jaypee Nigrie Super Thermal Power Plant in Singrauli, Madhya Pradesh, where two streams, with a total throughput of 3840 m^3/day, are treated to protect the high pressure boilers in the power-generating facilities. A tubular membrane for removing silica has also been tested using chemical polishing wastewater (oxide-CMP wastewater from a wafer factory, Taiwan). In this project, two inside-out tubular TiO_2/Al_2O_3 composite membranes with a MWCO of 95,000 Da were used to reduce the high concentration of colloidal silica (initial concentration 1316 mg/L of SiO_2) [31]. Due to the operating parameters of RO, this technique is not used to remove silica. Furthermore, UF, ion exchange, and electrocoagulation constitute an ideal pretreatment stage to protect RO from silica scaling [39,40].

Table 1. Metalloids removal from industrial wastewater.

Element	Technology	Basic Process Parameters	Results	Ref.
As	NF	Pilot-scale, membrane Dow/FilmTec NF90 with MWCO 100–200 Da, transmembrane pressure 5–20 bar, flow rate 1.2–3.2 L/min, As concentration 100–200 µg, other ions 10–2000 mg/L	Rejection: As(V) 98%, $SO_4{}^{2-}$ 95%, F^- 87%, and $NO_3{}^-$ 76%	[15]
	NF	pilot-scale; membrane NF-300 (Osmonics Inc), TFC polyamide membrane with MWCO 180 Da; operating pressure 7 bar; aqueous feed composition: 180 µg As(V)/L, 5 mg F/L and 84 mg HCO_3/L, and pH 8	Rejection: As(V) 93%, $HCO_3{}^-$ 89% and F^- 85%	[16]
	NF	NF90-4040 (Polyamide Thin-Film Composite (TFC)); operating temperature 28 °C, operating pressure 7 bar; aqueous feed composition: 500 µg/L As	Arsenate removal in 94%	[17]

Table 1. *Cont.*

Element	Technology	Basic Process Parameters	Results	Ref.
As, Se	NF	PA-CSBF4 (C-SBF content 40 mg), permeate flux 444 L/m^2 h, transmembrane pressure 0.5 bar; aqueous feed composition: pH = 7.0, arsenite and selenite concentration 100 µg/L, NaCl 0.01 mol/L; regenerating agent: NaOH (pH = 9)	Rejection: As(lll) 99%, Se (selenite and selenite) 98%	[22]
	NF	TFC membrane containing 50 wt% of P[MPC-co-AEMA], aqueous feed composition: 1 mg/L of As and Se ions, pH—7.5, 8.0 and 8.6, transmembrane pressure 10 bar PWP of 8.5 LMH/bar	Rejection: SeO_3^{2-} 98.2%, SeO_4^{2-} 99.1% and $HAsO_4^{2-}$ 99.8%	[25]
B	Multistage RO	Seawater desalination using CDBR process equipped with commercial RO or BF membranes, seawater composition: 35,000 ppm (mainly Na^+, Cl^-, Ca^{2+}, Mg^{2+}) OPD 56.6 bar energy consumption—2.70 kWh/m^3	Reduction: boron—0.5 ppm, salts -100 ppm, water recovery 65–75%	[33]
Si	Adsorption/UF	Brackish water; continuous stirred tank reactor; UFP-30-C-4A hollow fiber (MWCO 30,000 Da); residence time—15 min; agglomerates: iron oxy/hydroxide, adsorbent dosage up to 2 g/L	Rejection Si 93% for 20 mg/L and 67% for 60 mg/L	[37]
	Ultrafiltration/UF	HFS 60 Silica (Pentair X-Flow, MWCO 10,000 Da); Two streams totalling 6000 m^3/day	Rejection Si > 90%	[38]
	Tight UF	Inside-out tubular TiO_2/Al_2O_3 composite membranes (MWCO 95 Da); total solid content in oxide-CMP wastewater: 1333 mg/L (SiO_2 1316 mg/L) and pH 9.18, NTU 110; ORP 50.2 mV	Membrane cleaning Removal Si > 90%	[39]

3. Removal of Heavy Metals

3.1. Chromium (Cr)

Chromium in wastewater occurs in the third or sixth oxidation state. Depending on the degree of oxidation, different purification methods are used. This is due, among other things, to the fact that Cr(VI) are more dangerous and, therefore, much more thorough methods of removal or reduction to oxidation state three are required. Furthermore, it should be kept in mind that hexavalent chromium is in the form of chromate and dichromate anions (CrO_4^{2-} (or $HCrO_4^-$) and $Cr_2O_7^{2-}$, respectively), while trivalent chromium is in the form of cations Cr^{3+}. Cr(VI) is more dangerous precisely due to the oxyanions formed, which show high mobility and reactivity [41]. Chromate effluents are effluents generated by the textile, tannery, and electroplating industries. These effluents usually have a high concentration of pollutants, including a number of additional substances, such as surfactants, salts, and oil. Although chromium effluents are a major problem, in recent times, most of the work on membrane filtration techniques has focused on model solutions, often for single chromium ions. Although these results are promising, no large-scale studies have been conducted. For example, compared to a commercial RO membrane, the membrane proposed in the work [41], based on a branched poly(acryloyl hydrazide) star polymer with multiple amine groups, allowed an increase in Cr(VI) rejection from 55 to 99.5% at pH 3 and showed a higher regeneration capacity. However, the potential for the use of membranes in the treatment of chromium wastewater should be seen as an opportu-

nity, replacing traditional methods such as adsorption [42], also through hybrid solutions that combine several separation techniques. For example, Mousazadeh et al. [43] have proposed a coupled electrochemical-physical process that includes iron electrocoagulation, filtration, and sedimentation as pretreatment steps before Cr(VI) removal using the FO process. In addition to wastewater treatment processes, membrane separation techniques related to chromium ions are also successfully proposed for groundwater remediation, for example, using RO with chromium removal of 98.38% [44] or based on FO [45] and NF [46], RO/NF [47] processes. Recent works, based on real solutions, are summarized in Table 2.

3.2. Cobalt (Co)

The demand for the removal of Co(II) ions from industrial wastewater is very high because of their presence in frequently used lithium ion batteries and in the metallurgical industry. As a result of the high price of this metal, solutions are being sought to prevent its reflux into production streams. For example, Chen et al. demonstrate the potential for using FO to concentrate and reflux cobalt in a lithium battery plant [48]. In this solution, Co-based FO draw solute was obtained from lithium-ion battery waste, and this solute allowed for Co-containing wastewater purification. Cobalt may also be present in radioactive wastewater. Therefore, several studies are being conducted to test the feasibility of its removal by membrane techniques. For example, it was confirmed that high retention of Co(II), Sr(II) and Cs(I) can be obtained (99.6, 99.7 and 97%, respectively) based on filtration with MoS_2@NH_2-UiO-66-TFNi membrane [49]. Furthermore, in the case of Co recovery, micellar-enhanced UF was prosed with the support of two surfactants sodium dodecyl sulfate (SDS) and sodium oleate (SO) with a maximum retention of 99.95 and 99.99%, respectively [50].

3.3. Nickel (Ni)

Because of its widespread use, including in the production of stainless steel and in electroplating processes, the need to remove it from wastewater generated during its processing should be considered. Like other heavy metals, the metal is inert to the environment. Exposure to nickel can cause health problems such as skin irritation, asthma, and conjunctivitis, and in large amounts can cause cancer. Several methods such as precipitation, ion exchange, adsorption, electrochemical processes, and membrane techniques are proposed to remove nickel from solutions. The literature indicates that it is possible to effectively remove chromium ions from model aqueous solutions by FO using composite zeolite hollow fiber [51] and polydopamine/metal organic framework thin film nanocomposite membranes [52] or with multi-charged metallic complexes as draw solutes [53] or in the hybrid process NF with electrocoagulation [54] or NF alone with membrane modified with curcumin boehmite nanoparticles [55]. Moreover micellar or polymer-enhanced ultrafiltration is proposed for the removal of Ni(II) ions with the support of sodium dodecyl sulphate as surfactants [56] and poly(sodium acrylate) as polymers [57], respectively, as well as membrane filtration using polyethersulfone/α-zirconium phosphate (PES/α-ZrP) flat-sheet nanocomposite ultrafiltration membranes [58]. Despite numerous reports in the literature studying the possibility of removing nickel ions from aqueous solutions in recent years, there is no description of the work that confirms the feasibility of applying membrane filtration in real systems, as presented in Table 2.

3.4. Copper (Cu)

Copper is widely used in industries that include metal finishing, electroplating, plastics, and etching. As a consequence, copper ions are also expected in industrial effluents. Due to the toxic nature of copper, this effluent must be treated before being discharged into the environment. As with other heavy metals, several physicochemical methods are proposed for this purpose, including adsorption, ion exchange, extraction, and membrane techniques [59]. Most of the recent literature on Cu removal by membrane filtration techniques is based on model solutions. As presented by researchers, it is possible to obtain

a high Cu retention of Cu using polyethylenimine (PEI) cross-linked P84 NF membranes (>90%) [60], NF and FO process with piperazine/polyethyleneimine (PIP/PEI) membranes (95 and 99%, respectively) [61], NF like-forward osmosis (99.4%) [62], FO (95%) [63], RO (>90%) [64], NF (>90%) [65]. Moreover, with the support of Keggin polyoxometalates, the UF process allows one to obtain maximum metal retention at the level of 99% for Cd and Cu [66]. An interesting example of Cu(II) ion separation, together with an indication of the differences in copper salt (sulfate and chloride), is the solution presented in the paper [67]. Based on new nanocomposite membranes prepared by interfacial polymerization of polyethyleneimine (PEI) and trimesoyl chloride (TMC) with cellulose nanoparticles, the authors achieved a high degree of removal of toxic heavy metal ions ($CuSO_4$ 98.0%, $CuCl_2$ 96.5% and $PbCl_2$ 90.8%). Significantly lower retention values were obtained in the work, despite the modification of membranes with nanoparticles (L-cysteine functionalized POSS NP polyether-imide-thin film nanocomposite NF membrane), despite the modification of membranes with nanoparticles [68]. The maximum rejections for Na_2SO_4, $Pb(NO_3)_2$, $CrSO_4$, and $Cu(NO_3)_2$ were 84, 83, 81 and 79%, respectively. Examples of the applicability of membrane techniques for the removal of copper ions from real solutions are summarized in Table 2, mainly in the form of multi-ion mixtures.

3.5. Zinc (Zn)

In recent years, only a few papers have appeared on the removal of zinc ions. This seems to be due to the fact that most of the current research is focused on strategic metals. However, it should be borne in mind that zinc effluent is a serious environmental problem and research should be carried out on its removal or possible return to the process. As presented in [69], the NF process could be considered in Zn separation. NF AFC membranes allowed 98% rejection for sulphate and nitrate zinc(II) salts, while AFC 30 was able to efficiently remove of Zn only as $ZnSO_4$ (R = 98%, and up to 70% for $Zn(NO_3)_2$).

3.6. Cadmium (Cd)

As with the metals previously discussed, cadmium is removed from aqueous media, among other things, by membrane techniques. Because of the strong toxicity of this compound, very efficient removal processes are required. In recent years, the authors have demonstrated the great potential of membrane filtration in this field. In the case of model solutions, it has been shown that cadmium can be removed by FO, NF, and UF. For this purpose, it is proposed to use a synthetic thin-film nanocomposite FO membrane modified by graphene oxide and polyethylene glycol (retention of Cr, Cd, and Pb 98.3, 99.7 and 99.9%, respectively) [70] or by adding titanium nanotubes and magnetite oxide hybrid nanoparticles (TNT–Fe_3O_4) in polysulfone membrane (R > 98%) [71] and cellulose acetate NF membranes (R = 98% [72] or in-situ Cu NP enhanced ceramic-supported polymeric composite NF membrane enhanced with Cu NP (R = 95.5%) [73]. In addition, the polymer enhanced ultrafiltration (PEUF) process was successfully applied (the retention reached 100%) for the removal of Cd using soluble polymers, chitosan, polyvinyl alcohol, and polyacrylate sodium [74] or the selective retention of Cd-Ni ions from aqueous solutions [75].

3.7. Mercury (Hg)

Mercury wastewater is a waste with such a high toxic load on human health, life, and the environment that industry is obliged to strictly control it. Therefore, efficient methods of Hg removal are still being sought, including through the development of membrane techniques. For example, micellar enhanced ultrafiltration (MEUF) with sodium dodecyl sulphate (SDS) using a polyacrylonitrile membrane allows the rejection of Hg up to 96.83% [76] or with SDS and cetylpyridinium chloride (As, Hg retention 95%) [77], while the NF process with L-cystine/L-cysteine impregnated with L-cystine/L-cysteine shows very high retention (99.99%) and can effectively reduce the Hg(II) concentration from 10 ppm to 0.18 ppb, thus below the acceptable limits in drinking water (2 ppb) [78].

Similar results are obtained in the pyrite (FeS_2)-supported UF process (R ≈ 100%), with adsorption on pyrite and membrane filtration [79]. The process with hybrid membrane from whey protein fibrils and activated carbon is less efficient. Hg and Cr retention is equal to 81 and 57%, respectively [80].

3.8. Lead (Pb)

Lead must also be virtually completely removed from wastewater as a result of its strong toxic effects. In the case of model solutions, it has been shown that membrane techniques can meet this challenge. As mentioned above, the use of the FO process is possible to obtain 98% or Pb rejection [71]. Lower efficiency (R > 90%) is provided by the NF process using a cross-linked polyethylenimine (PEI) membrane P84 membrane [60]. The NF process with a thin film nanocomposite membrane incorporated UiO-66-NH_2 [81] and the biosorption hybrid process [82] can also be also used. Furthermore, using the mixed matrix UF membrane, there is a possibility to remove up to 95% of Pb and 94% of Cd [83] or 94.8% in the UF process with the support of extracellular polymeric substances [84].

Table 2. Heavy metals removal from industrial wastewater.

Element	Technology	Basic Process Parameters	Results	Ref.
Cr	RO diafiltration	Tannery industry: Real sludge from TAMEG-Rouiba-SPA—a Leather Industry, Rouiba, Algeria, conc. in mg/L: Cr 50, Fe 4.64, Ni 0.27, Cu 1.54, B 0.12, Ca 81, K 79.8, Mg 67.2, Na 259, P 0.36, S 58.3, Si 9.7, Sr 0.97. RO membrane: SW30 (polyamide thin film composite), DOW Chemical Company Diafiltration membrane: polyethersulfone (PES) MF membrane top-coated with a chitosan layer	RO: More than 95% rejections for all inorganic salts (99.2% for Cr). Diafiltration: Recovery of Cr (III) in RO retentate with the addition of acidified water to pH 3.6. Retain 97% Cr(III), with selectivity for NH_4^+ (4.2), Cl^- (5), K^+ (12.9), Na^+ (14.6) and Mg^{2+}, Ca^{2+}, S^{2-} (>45), due to Cr (III) adsorption on the chitosan membrane and high permeability of other ions. Desorption of Cr(III) at pH 2: recovery of 92.5% Cr(III) from RO concentrate. The solution can be reused in the tannery process.	[85]
	NF, RO	Tannery industry: Real sludge from TAMEG-Rouiba-SPA—a Leather Industry, Rouiba, Algeria NF: NF270 and NF90 membranes, RO (BW30 and SW30) and polyethersulfone (PES) MF membrane coated by chitosan	Best option: RO in the first step with SW30, second step selective recovery of Cr(III) in the second step from the retentate using a modified chitosan membrane (permeate with <0.01% Cr). New chitosan membrane: Cr removal >99%, more than 8 and 6 times higher compared to monovalent cations (Na^+ and K^+) and divalent cations (Mg^{2+} and Ca^{2+}), respectively.	[86]
	UF	Tannery industry: Sludge from the tannery industries, Site-2, Unnao, UP. UF: polyvinylidene fluoride/titanium dioxide solar active photocatalytic membrane	The UF membrane has an excellent rejection and reduction ability from Cr(VI) to Cr(III): 97.59% and 91.73% for the model solution and 90% and 85% for real wastewater.	[87]
	RO, electro-cogulation	Leather industry wastewater from Al-Nahrawan, Iraq, conc. in g/L: Cr(III) 1.6. Hybrid process: electrocoagulation (EC) and RO (feed solution electrolyte after EC, 0.12 g/L of Cr)	Rejection of Cr 88.8% after EC and 99.89% after EC/RO; recovery percentage ranged between 8.03 and 25.31%.	[88]
	UF, NF, RO, ED	Tannery industry: Sludge from the leather company in Fujian, concentration in mg/L: Ca 250–280, Mg 100–200, Na 1500–1600, chroma 600–1000 UF: PVC, PES membranes, cut off 65, 100, 150 kDa	Process flow chart: flocculation, sedimentation, UF, NF, RO, and ED. Flocculation-UF process with 150 kDa PVC membrane to remove the suspended solids and macromolecular NF process to improve recovery rate, ED for the desalting stage.	[89]

Table 2. *Cont.*

Element	Technology	Basic Process Parameters	Results	Ref.
	FO	Wastewater from the processing of Acrylonitrile Butadiene Styrene/Polycarbonate plastics, conc. in g/L: Cr(VI) 50.9<br>FO: Aquaporin Inside membrane hollow fibre FO (AIM™ HFFO) modules, DS: 1 M NaCl	Rejection of Cr(VI) up to 99.74%, due to electrostatic repulsion between the negative charged membrane surface and the anions ($HCrO_4^-$ and $Cr_2O_7^{2-}$).<br>The membrane material is damaged due to the oxidizing character of Cr(VI) and should be modified.	[90]
	RO	Electroplating wastewater: from BIA Kunststoff- und Galvanotechnik GmbH & Co. KG, conc. in g/L: Cr(III) 0.77, $B(OH)_3$ 7.18, SO_4^{2-} 7.12.<br>RO: polyamide thin-film composite Flmtec SW30-2540, DuPont	Rejections of boric acid 93.8%, Cr(III) 99.9%, sulfate 99.6% for sulfate with 8.4 g/L Cr(III) in RO retentate.<br>Hull cell electroplating tests showed that the deposition of cold-hued chromium layers is possible directly from the retentate solution.	[91]
	FO	Sewage sludge: model based on real effluents, conc, in mg/L Cr(VI) 10, COD ($C_6H_{12}O_6 \cdot H_2O$) 500, TP (KH_2PO_4) 20, NH_4Cl 20<br>FO: with TFC membrane, DS: temperature-sensitive hydrogels based on sodium alginate	High removal in the process is obtained: Cr(VI): 96.9%–97.4%, COD: 97.1%–97.4%, TP: 97.7%–99.6%, and NH_4^+Cl: 76.8%–77.9% with high water flux.	[92]
Cr, Sb	FO	Printing and dyeing factory: conc. in wastewater, in ppb total Cr 23.93, Sb 0.43, aniline 46.03<br>FO: with a flat thin–film composite (TFC) membrane, draw solution (DS): 1.5 wt.% LiCl.	Rejection of Cr, Sb, and aniline, after 10 h of FO operation, 99, 98, 99.5%, respectively. Cr was classified mainly as Cr(VI).	[93]
Ni, Cu, Zn, Cd, U, Pb, Th, K	RO	Mining industry: leaching solution of phosphogypsum from the Al-Qaim fertilizers complex at the Al-Anbar government	RO removal of Ni, Cu, Zn, Cd, U, Pb, Th, K,) with maximum rejection: 76.6, 77.5, 80.2, 81, 90.9, 92.9, 93.9%, respectively.	[94]
Sb, As, Ni, Zn, Fe	RO	Mining industry: wastewater treatment plant: Costerfield, Mandalay Resources Ltd., Victoria, Australia. Sludge from underground gold-antimony mining, processing plant, water treatment plant, evaporation, and tailing storage facilities, max. conc. in the feed in mg/L: Sb 50.2, As 0.047, Ni 0.03, Zn 0.104, Fe 1.19, Cd 0.0001, Cr 0.001, Cu 0.004, Pb 0.002<br>RO: 96 polyamide membranes DOW™ FILMTEC™ BW30-440i	RO efficiency, reduction in the concentration of Sb, As, Ni, Zn, Fe by 95, 66, 82, 48 and 10%, respectively, in the RO permeate compared to the feed water.<br>Membranes, due to their tendency to fouling and damage in harsh conditions, require pre-cleaning of the feed solution.	[95]
Cr, Pb, Cd, As, Ni, Sb	RO, NF	Municipal sewage treatment: surface water in the Democratic Republic of Congo; conc. in ppm Cr 0.06, Pb, Cd, As, Sb < 0.05, Ni 0.03<br>RO: polyamide urea X-20 membrane, NF: NF90 and NF 270 membranes from Lenntech Water Treatment Solutions	RO removal of Cr(III), Pb(II), Cd(II), As(III), Ni(II), Sb(III) with a rejection of 99.2, 98.8, 98.6, 99.2, 98.4, 98.8%, respectively.<br>NF removal lower than RO, with a rejection of 98.2, 76.9, 92.3, 52.5, 97.8, 64.1%, respectively.<br>NF is the best option for the removal of heavy metals from low-concentration wastewater, while RO is for a very high concentration.	[96]
Cu, Zn, Ag, Pb	RO	San Pedro Porphyry Deposit in the San Rafael Massif,<br>RO commercial membrane	Rejection above 90% for Cu, Zn, Ag, and Pb. Metal osmotic differentiation at low temperatures favored atypical Ag-bearing ore paragenesis.	[97]
Cu	RO, FO	Acid mine drainage (AMD) formed by the natural oxidation of sulfide minerals, such as pyrite,<br>NF: TFNC membrane<br>FO: 1 M ammonium dihydrogen phosphate and ammonium sulfate as draw solutions.	The NF process showed a high copper concentration capacity (0.6 to 2.4 g/L) and a good total rejection of species (~82%) and a high water recovery of 80% in FO.<br>The combined NF-solvent extraction with LIX 84-IC resulted in a high recovery of water and Cu from AMD.	[98]

Table 2. *Cont.*

Element	Technology	Basic Process Parameters	Results	Ref.
Cr, Fe, Ni, Cu, Zn, Pb, Au	Electrochemical-osmotic (EOS) system with NF membranes	Electroplating wastewater was collected from UniMetal Surface Finishing Company, Waterfield, CT, USA, conc. in mg/L: Cr 11.31, Fe 9.53, Ni 63.42, Cu 312.54, Zn 24.62, Pb 2.81, Au < 1<br>EOS: polyelectrolyte multilayer NF membranes	Water/salt selectivity of the PMNF membrane up to 25.1 L/mol, water production rate of 6.06 L/m^2h and the power density of 1.18 mW/cm^2 by treating synthetic electroplating wastewater, 2.63 and 1.21.	[99]
Fe, Zn, Na, As, Ca, Cu, Ni, Mn	NF	Hydrometallurgical copper industry, conc. in mg/L: Fe(II) 6390, Fe(III) 4566, Zn 722, Na 649, As 508, Ca 500, Cu 230, Ni 98, Mn 60<br>NF: extreme acid-resistant Duracid membrane.	Metal rejections of more than 90%, H^+, could permeate across the membrane.	[100]
Hg	UF, adsorption	Industrial wastewater from industrial site in California, conc. in ppm Hg 0.05, Na 357, Mg 26, Ca 52, K17<br>Three-step process: primary filtration using a PVDF membrane to remove particulates; UF membrane to remove mercury sulfide NPs, and adsorption with thiol-functionalized membranes to remove dissolved mercury	The UF membrane was able to effectively remove mercury sulfide nanoparticles from wastewater, thiol membranes were also found to be effective at removing dissolved mercury, with adsorption efficiencies of up to 97% observed over a 20 h period.	[101]
Cr, Pb, Fe, Zn, Si	MF/RO	Wastewater treatment plant located in an industrial area known as an "Organized Industrial Zone" (OIZ), conc. in mg/L Cr 1.5, Ob 1.5, Cd 0.1, Fe 10, Cu 3, Zn 5, Hg 0.05<br>RO membranes (BW30, HP and LE) for chemical treatment and ceramic microfiltration (MF) as pretreatment steps.	The removal efficiencies for various contaminants in the wastewater ranged from 40 to86.3% for chemical oxygen demand (COD), 97.6 to 99% for S ions, 69.2 to 94.9% for Cr ions, 89.3 to 100% for Pb ions, 66.3 to 98.2% for Fe ions, 97.5 to 99.7% for Zn ions, 95.1 to 99.5% for Si ions, and 79.1 to 100% for total phosphorus.	[102]
Pb, Zn, Cd	UF, RO	Pb-Zn smelter wastewater from the smelter in Zhuzhou, China, conc. in mg/L Ca 600–900, Zn 1.5–5, Fe 0.4–0.7, Cu 0.1–0.5, Pb 0.2–1.2, Hg 0.01–0.1, Cd 0.3–0.9, As 0.3–0.5, Ba 0.025–0.035, Sr 0.2–0.4<br>UF: PVDF membrane<br>RO: polyamide thin film composite membrane	The removal of Cd(II) is nearly 100% at pH 5.5, while the rejection of Pb(II) is less than 60% and the rejection of Zn(II) is also less than 60%. When the pH is increased to 7.0, the removal rate of Pb(II) approaches 100%, while the removal rates of Cd(II) and Zn(II) are lower.	[103]
Pb, Zn		Wastewater for a smelting plant located in the central-south of China, conc. in mg/L Ca 600–900, Zn 1.5–5, Fe 0.4–0.7, Cu 0.1–0.5, Pb 0.2–1.2, Hg 0.01–0.1, Cd 0.3–0.9, As 0.3–0.5, Ba 0.025–0.035, Sr 0.2–0.4<br>Several steps process: 1st coagulation-flocculation-sedimentation (CFS), 2nd multi-media filtration (MMF) as a pretreatment for UF, 3rd UF as a pretreatment for RO	The process had a wastewater recovery rate of 87.4% or higher, with salt, heavy metal ions, and conductivity rejection rates of 97% or higher. The resulting reclaimed water had a conductivity of 220 µS/cm.	[104]
Zn	Adsorption, RO	Wastewater from the Esfarayen Steel Industrial Complex, Malysia, conc. in mg/L Cu 0.83, Mn 1.56, Zn 4.02, Fe 23.30, Al 1.46 adsorption with activated carbon as pretreatment for RO.	Removal efficiencies of 98.1% for dissolved solids, 97.4% for electrocoagulation, 100% for Zn and 95.3% for turbidity. Additionally, the system was found to be resistant to high concentrations of contaminants, with removal efficiencies of more than 90%.	[105]

4. Platinum Group Metals

Platinum group metals (PGMs), due to their unique properties (high melting points, high heat resistance, high corrosion resistance, and strong catalytic activity), are used in key industry sectors, for example, as catalysts in the automotive industry, petroleum refining, industrial chemical production (nitric acid, ammonia, silicones, and petrochemical feedstocks) [106,107]. In addition, the production of fuel cells, novel magnetic storage media and catalysts are sectors that are developing at a faster rate and are resulting in a significant increase in the use of PGMs [108]. The depletion of natural metal resources

and the growing environmental requirements force the industry to look for solutions that enable not only the effective extraction of metals from mineral deposits, but also recycling from waste streams [108–110]. Spent automotive converters are considered to be an important source of Pt, Pd and Rh, and hydrometallurgical operations such as leaching from solids or extraction from heavy leaching solutions allow them to be efficiently recovered and recycled. Due to its physicochemical properties, the acquisition and concentration of PGM requires treatment with acidic oxidizing agents, aqua regia, or concentrated alkaline solutions. As a result, further stages of metal separation significantly limit the wide selection of techniques for solvent extraction, electrowinning, precipitation, or ion-exchange. Other techniques show low application potential or are part of a hybrid system (due to low efficiency and selectivity) [111,112]. Recovery of PMs using supported liquid membranes has been the subject of broad investigation by numerous researchers throughout the world. The latest literature reports indicate that more and more articles are dealing with the subject of obtaining PGM separation from waste leaching solutions. For example, Noah et al. [113] have proposed an emulsion liquid membrane (ELM) for the selective extraction of palladium from electroplating wastewater. In this process, the main components of the membrane were as follows: CYANEX 302 in kerosene as a carrier, 1 M thiourea in 1 M H_2SO_4 of stripping agent as internal stripping phase, and span 80 as surfactant. The result using the real electroplating wastewater showed that almost 100% of Pd(II) was extracted selectively over chromium under these conditions. In another work, a hollow fiber-supported liquid membrane (HFSLM) with Aliquat 336 as a carrier was tested to recover Pd(II) from wastewater containing Cu(II) and Ni(II). It was shown, that at pH 2, using 0.5 M of thiourea mixed with 0.1 M HCl as a stripping agent and 100 mL/min of flow rate for both phases, the extraction and stripping of Pd (II) reached >99% and 87.09%, respectively [114]. In addition, high efficiency in Pd(II) removal, comparable to classical extraction, was obtained using pseudo-emulsion based hollow fibre strip dispersion (PEHFSD) [115]. In this process, a pseudoemulsion is an emulsion that temporarily forms between the organic and stripping phases, providing simultaneous extraction and reaction in a single hollow fiber contactor, thus removing Pd(II) removal in the continuous system. The invention of this system was the use of a non-commercial, but more effective and selective carrier (N-decyloxy-1-(pyridin-3-yl)ethaneimine). The tests conducted have shown that the new proposed extractant has greater potential for the extraction and stripping of Pd(II) than commercial extractants. Researchers have also proposed a polymer inclusion membrane process to recover valuable metal ions, but at a much lower concentration than that observed in leach solutions [116]. Few works have also concerned modification of the filter material, and the obtained membranes enabled the removal of Pd(II) by adsorption. The modification provided satisfactory rejection, while the selectivity was low even for Cu(II) and Ni(II) [117].

5. Perspectives

The retention values shown in Tables 1 and 2, as well as in text are presented in Figure 4, by using a method of graphically demonstrating the locality, spread, and skewness of groups of numerical data through their quartiles, the so-called box plot. In the case of outliers, they were plotted as single points outside the whiskers on the box plot. The analysis of the data indicates the existence of a scatter in the obtained results of the efficiency of membrane processes, which shows the great importance of the appropriate choice of separation method. One should also keep in mind the limiting factor of this analysis, namely the amount of experimental data acquired, which vary considerably depending on the metal under consideration. The analysis presented in the study of the performance of membrane separation techniques for the treatment of industrial wastewater generally showed high efficiency for all metals. Metal retention rates are high, often reaching 100%, as presented in Figure 4. However, in the case of 100% efficiency, it is necessary to take into account the imperfections of the measurements made, including the limit of determination of the ions in question by the authors of the cited works. However, it should be noted that

even if this efficiency is not as high as declared, the level of impurities in the permeate (at the limit of quantification) is so low that it meets environmental requirements. The method should therefore be regarded as 100% effective, despite the known limitations.

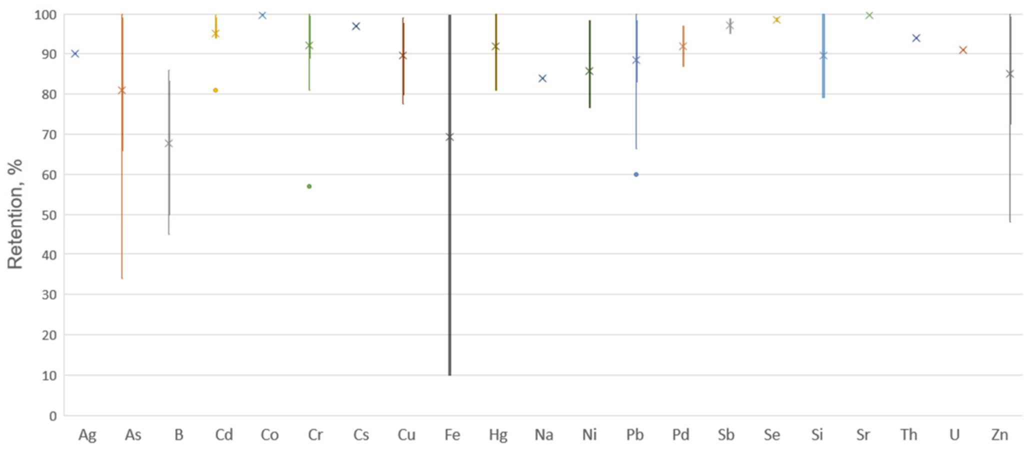

Figure 4. Box plot of data from Tables 1 and 2 (metal removal efficiency).

An analysis of the literature clearly confirms the importance of the membrane's structure, including its composition and manufacturing method, in determining its permeability and selectivity [118]. The challenges of material selection when using membrane techniques appear to be the most significant. The choice of material has an impact on the membrane's separation properties. In addition to the use of a range of polymers (cellulose acetate (CA), polysulfone (PS), and polyethersulfone (PES)) with suitable wetting and pore-size properties or ceramic membranes, which improve the membrane strength to a significant extent, there is also the challenge of focussing on the functionalization of membrane surfaces with a range of components. The membrane based on the metal structure [119–121], nanoparticles [122,123], or other functional groups [124] can be mentioned here. The most important factors mentioned in this paper to improve the efficiency of the separation process are shown in Figure 5. The next big challenge is the development of hybrid solutions. When a number of techniques enter a single process line, the efficiency of wastewater treatment can be improved. A summary of these already proposed solutions is shown in Figure 5. This research direction seems to have great potential, as it becomes possible to exploit the potential of different separation methods.

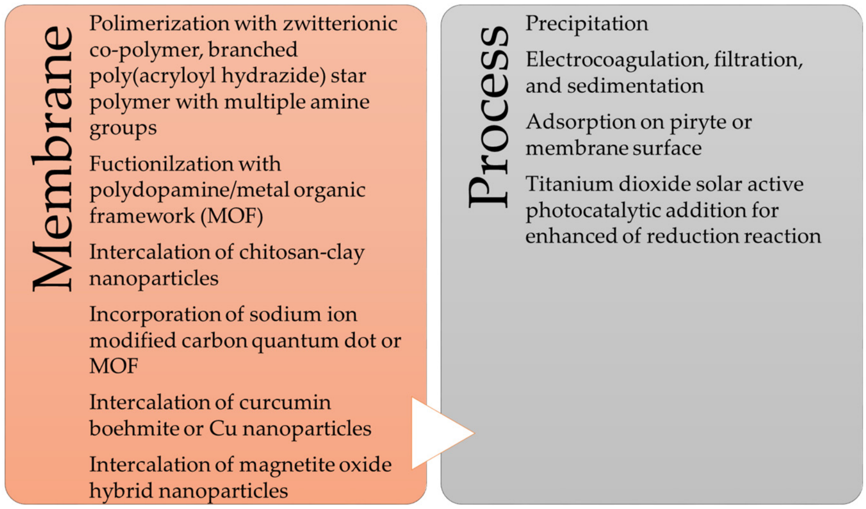

Figure 5. Trends in the development and modification of membranes and membrane techniques.

6. Conclusions

The presented analysis of recent literature shows the high potential of using membrane techniques to treat industrial wastewater and return waste streams back into the process. This fits in with recent trends towards a circular and zero-waste economy. The summary clearly demonstrates the need for further work to improve the efficiency and selectivity of membrane techniques. It seems that a greater emphasis on research on real wastewaters should lead to greater industrial interest in such solutions. Therefore, work is needed to develop new membrane materials that are durable enough to withstand the harsh conditions of industrial wastewater treatment plants. Current directions for the modification of membranes by copolymerisation, the addition of MOF, nanoparticles and other functional groups are presented. Last but not least, attempts should be made to optimise the removal of metal ions from aqueous solutions, not only by finding the best process conditions, but also by looking for hybrid solutions. The research presented here shows that the combination of several purification techniques gives the best results. Thus, it should be noted that, despite the maturity of membrane methods, scientists still have challenges to meet with industrial requirements.

Author Contributions: Conceptualization, K.S. and K.W.; writing—original draft preparation, K.S. and K.W.; writing—review and editing, K.S. and K.W. All authors have read and agreed to the published version of the manuscript.

Funding: This research was supported by the Polish Ministry of Science end Education (grants No. 0912/SBAD/2210, 0912/SBAD/2200).

Institutional Review Board Statement: Not applicable.

Data Availability Statement: Not applicable.

Conflicts of Interest: The authors declare no conflict of interest.

References

1. Papaioannou, E.H.; Mazzei, R.; Bazzarelli, F.; Piacentini, E.; Giannakopoulos, V.; Roberts, M.R.; Giorno, L. Agri-Food Industry Waste as Resource of Chemicals: The Role of Membrane Technology in Their Sustainable Recycling. *Sustainability* **2022**, *14*, 1483. [CrossRef]
2. Staszak, K.; Wieszczycka, K. Membrane applications in the food industry. *Phys. Sci. Rev.* **2022**. [CrossRef]
3. Brinkmann, T.; Santonja, G.G.; Yükseler, H.; Roudier, S.; Sancho, L.D. *Best Available Techniques (BAT) Reference Document for Common Waste Water and Waste Gas Treatment/Management Systems in the Chemical Sector*; Publications Office of the European Union: Luxembourg, 2016; ISBN 9789279619960.
4. Ahmed, S.F.; Mehejabin, F.; Momtahin, A.; Tasannum, N.; Faria, N.T.; Mofijur, M.; Hoang, A.T.; Vo, D.-V.N.; Mahlia, T.M.I. Strategies to improve membrane performance in wastewater treatment. *Chemosphere* **2022**, *306*, 135527. [CrossRef] [PubMed]
5. Qasem, N.A.A.; Mohammed, R.H.; Lawal, D.U. Removal of heavy metal ions from wastewater: A comprehensive and critical review. *Npj Clean Water* **2021**, *4*, 36. [CrossRef]
6. Saleh, T.A.; Mustaqeem, M.; Khaled, M. Water treatment technologies in removing heavy metal ions from wastewater: A review. *Environ. Nanotechnol. Monit. Manag.* **2022**, *17*, 100617. [CrossRef]
7. Kabay, N.; Shirazi, M.M.A.; Güler, E.; Bryjak, M. Grand Challenges in Membrane Modules and Processes. *Front. Membr. Sci. Technol.* **2022**, *1*, 4. [CrossRef]
8. Guiver, M.D. Field Grand Challenge for Membrane Science and Technology. *Front. Membr. Sci. Technol.* **2022**, *1*, 2. [CrossRef]
9. Fatoki, J.O.; Badmus, J.A. Arsenic as an environmental and human health antagonist: A review of its toxicity and disease initiation. *J. Hazard. Mater. Adv.* **2022**, *5*, 100052. [CrossRef]
10. Issa, N.B.; Rajaković-Ognjanović, V.N.; Marinković, A.D.; Rajaković, L.V. Separation and determination of arsenic species in water by selective exchange and hybrid resins. *Anal. Chim. Acta* **2011**, *706*, 191–198. [CrossRef]
11. Alka, S.; Shahir, S.; Ibrahim, N.; Ndejiko, M.J.; Vo, D.-V.N.; Manan, F.A. Arsenic removal technologies and future trends: A mini review. *J. Clean. Prod.* **2021**, *278*, 123805. [CrossRef]
12. Siddique, T.A.; Dutta, N.K.; Choudhury, N.R. Nanomaterials Nanofiltration for Arsenic Removal: Challenges, Recent Developments, and Perspectives. *Nanomaterials* **2020**, *10*, 1323. [CrossRef]
13. Worou, C.N.; Chen, Z.-L.; Bacharou, T. Arsenic removal from water by nanofiltration membrane: Potentials and limitations. *Water Pract. Technol.* **2021**, *16*, 291–319. [CrossRef]
14. Vrijenhoek, E.M.; Waypa, J.J. Arsenic removal from drinking water by a "loose" nanofiltration membrane. *Desalination* **2000**, *130*, 265–277. [CrossRef]

15. Jadhav, S.V.; Marathe, K.V.; Rathod, V.K. A pilot scale concurrent removal of fluoride, arsenic, sulfate and nitrate by using nanofiltration: Competing ion interaction and modelling approach. *J. Water Process Eng.* **2016**, *13*, 153–167. [CrossRef]
16. Padilla, A.P.; Saitua, H. Performance of simultaneous arsenic, fluoride and alkalinity (bicarbonate) rejection by pilot-scale nanofiltration. *Desalination* **2010**, *257*, 16–21. [CrossRef]
17. Harfoush, M.; Mirbagheri, S.A.; Ehteshami, M.; Nejati, S. Arsenic removal from drinking water using low-pressure nanofiltration under various operating conditions. *Water Pract. Technol.* **2018**, *13*, 295–302. [CrossRef]
18. Ahmed, S.; Rasul, M.G.; Hasib, M.A.; Watanabe, Y. Performance of nanofiltration membrane in a vibrating module (VSEP-NF) for arsenic removal. *Desalination* **2010**, *252*, 127–134. [CrossRef]
19. Wong, W.; Wong, H.Y.; Badruzzaman, A.B.M.; Goh, H.H.; Zaman, M. Recent advances in exploitation of nanomaterial for arsenic removal from water: A review. *Nanotechnology* **2016**, *28*, 042001. [CrossRef]
20. Pérez-Sicairos, S.; Lin, S.W.; Félix-Navarro, R.M.; Espinoza-Gómez, H. Rejection of As(III) and As(V) from arsenic contaminated water via electro-cross-flow negatively charged nanofiltration membrane system. *Desalination* **2009**, *249*, 458–465. [CrossRef]
21. Stefaniak, J.; Dutta, A.; Verbinnen, B.; Shakya, M.; Rene, E.R. Selenium removal from mining and process wastewater: A systematic review of available technologies. *J. Water Supply Res. Technol.* **2018**, *67*, 903–918. [CrossRef]
22. Zeeshan, M.H.; Khan, R.U.; Shafiq, M.; Sabir, A. Polyamide intercalated nanofiltration membrane modified with biofunctionalized core shell composite for efficient removal of Arsenic and Selenium from wastewater. *J. Water Process Eng.* **2020**, *34*, 101175. [CrossRef]
23. He, Y.; Zhao, D.L.; Chung, T.-S. Na+ functionalized carbon quantum dot incorporated thin-film nanocomposite membranes for selenium and arsenic removal. *J. Memb. Sci.* **2018**, *564*, 483–491. [CrossRef]
24. He, Y.; Tang, Y.P.; Ma, D.; Chung, T.-S. UiO-66 incorporated thin-film nanocomposite membranes for efficient selenium and arsenic removal. *J. Memb. Sci.* **2017**, *541*, 262–270. [CrossRef]
25. He, Y.; Liu, J.; Han, G.; Chung, T.-S. Novel thin-film composite nanofiltration membranes consisting of a zwitterionic co-polymer for selenium and arsenic removal. *J. Memb. Sci.* **2018**, *555*, 299–306. [CrossRef]
26. Wan, P.; Yuan, M.; Yu, X.; Zhang, Z.; Deng, B. Arsenate removal by reactive mixed matrix PVDF hollow fiber membranes with UIO-66 metal organic frameworks. *Chem. Eng. J.* **2020**, *382*, 122921. [CrossRef]
27. Kumar, M.; RaoT, S.; Isloor, A.M.; Ibrahim, G.P.S.; Inamuddin; Ismail, N.; Ismail, A.F.; Asiri, A.M. Use of cellulose acetate/polyphenylsulfone derivatives to fabricate ultrafiltration hollow fiber membranes for the removal of arsenic from drinking water. *Int. J. Biol. Macromol.* **2019**, *129*, 715–727. [CrossRef]
28. Ahmad, A.; Rutten, S.; de Waal, L.; Vollaard, P.; van Genuchten, C.; Bruning, H.; Cornelissen, E.; van der Wal, A. Mechanisms of arsenate removal and membrane fouling in ferric based coprecipitation–low pressure membrane filtration systems. *Sep. Purif. Technol.* **2020**, *241*, 116644. [CrossRef]
29. López Zavala, M.Á.; Frías Bouchez, B. Montmorillonite-perlite-iron ceramic membranes for the adsorption/removal of As(III) and other constituents from surface water. *Ceram. Int.* **2022**, *48*, 31695–31704. [CrossRef]
30. Tagliabue, M.; Reverberi, A.P.; Bagatin, R. Boron removal from water: Needs, challenges and perspectives. *J. Clean. Prod.* **2014**, *77*, 56–64. [CrossRef]
31. Van der Bruggen, B.; Vandecasteele, C. Distillation vs. membrane filtration: Overview of process evolutions in seawater desalination. *Desalination* **2002**, *143*, 207–218. [CrossRef]
32. Kürklü, S.; Velioğlu, S.; Ahunbay, M.G.; Tantekin-Ersolmaz, S.B.; Krantz, W.B. A novel energy-efficient concurrent desalination and boron removal (CDBR) process. *Desalination* **2017**, *423*, 79–94. [CrossRef]
33. Kayaci, S.; Tantekin-Ersolmaz, S.B.; Ahunbay, M.G.; Krantz, W.B. Technical and economic feasibility of the concurrent desalination and boron removal (CDBR) process. *Desalination* **2020**, *486*, 114474. [CrossRef]
34. Neo, J.G.; Japip, S.; Luo, L.; Chung, T.-S.; Weber, M.; Maletzko, C. Hydroxyl-terminated poly(ethyleneimine) polymer enhanced ultrafiltration for boron removal. *Sep. Purif. Technol.* **2019**, *222*, 214–220. [CrossRef]
35. Kumar, R.; Ahmed, M.; Bhadrachari, G.; Al- Muqahwi, S.; Thomas, J.P. Thin-film nanocomposite membrane comprised of a novel phosphonic acid derivative of titanium dioxide for efficient boron removal. *J. Environ. Chem. Eng.* **2021**, *9*, 105722. [CrossRef]
36. Çermikli, E.; Şen, F.; Altıok, E.; Wolska, J.; Cyganowski, P.; Kabay, N.; Bryjak, M.; Arda, M.; Yüksel, M. Performances of novel chelating ion exchange resins for boron and arsenic removal from saline geothermal water using adsorption-membrane filtration hybrid process. *Desalination* **2020**, *491*, 114504. [CrossRef]
37. Shemer, H.; Melki-Dabush, N.; Semiat, R. Removal of silica from brackish water by integrated adsorption/ultrafiltration process. *Environ. Sci. Pollut. Res.* **2019**, *26*, 31623–31631. [CrossRef]
38. Silica removal projects in India use hollow-fibre membranes. *Membr. Technol.* **2012**, *2012*, 6. [CrossRef]
39. Yang, G.C.C.; Li, C.J. Electrofiltration of silica nanoparticle-containing wastewater using tubular ceramic membranes. *Sep. Purif. Technol.* **2007**, *58*, 159–165. [CrossRef]
40. Sheikholeslami, R.; Bright, J. Silica and metals removal by pretreatment to prevent fouling of reverse osmosis membranes. *Desalination* **2002**, *143*, 255–267. [CrossRef]
41. Jo, J.H.; Shin, S.S.; Jeon, S.; Park, S.-J.; Park, H.; Park, Y.-I.; Lee, J.-H. Star polymer-assembled adsorptive membranes for effective Cr(VI) removal. *Chem. Eng. J.* **2022**, *449*, 137883. [CrossRef]
42. Filipowiak, K.; Wieszczycka, K.; Buchwald, T.; Nowicki, M.; Wójcik, G.; Aksamitowski, P.; Staszak, K. Reduction-adsorption of chromium(VI) by using IL-imprinted resin-innovative solution for water purification. *J. Mol. Liq.* **2021**, *343*, 116977. [CrossRef]

43. Mousazadeh, M.; Naghdali, Z.; Kabdaşlı, I.; Sandoval, M.A.; Titchou, F.E.; Malekdar, F.; Nasr, M.; Martínez-Huitle, C.A.; Lichtfouse, E.; Emamjomeh, M.M. Reclamation of forward osmosis reject water containing hexavalent chromium via coupled electrochemical-physical processes. *Environ. Technol.* **2022**. [CrossRef]
44. Karunakaran, A.; Chaturvedi, A.; Ali, J.; Singh, R.; Agarwal, S.; Garg, M.C. Response surface methodology-based modeling and optimization of chromium removal using spiral-wound reverse-osmosis membrane setup. *Int. J. Environ. Sci. Technol.* **2022**, *19*, 5999–6010. [CrossRef]
45. Pham, M.T.; Nishihama, S.; Yoshizuka, K. Removal of Chromium from Water Environment by Forward Osmosis System. *MATEC Web Conf.* **2021**, *333*, 04007. [CrossRef]
46. Fadhil, S. Sustainable chromium removal by nanofiltration membranes: Application of pore flow model. *Int. J. Environ. Sci. Technol.* **2022**. [CrossRef]
47. Fuoco, I.; Figoli, A.; Criscuoli, A.; Brozzo, G.; De Rosa, R.; Gabriele, B.; Apollaro, C. Geochemical modeling of chromium release in natural waters and treatment by RO/NF membrane processes. *Chemosphere* **2020**, *254*, 126696. [CrossRef]
48. Chen, R.; Qiu, F.; Meng, Q.-W.; Chung, T.-S.; Ge, Q. A cobalt-based forward osmosis draw solute synthesized from lithium-ion battery wastes for cobalt-containing wastewater purification. *Desalination* **2023**, *548*, 116279. [CrossRef]
49. Wang, J.; Wang, L.; He, M.; Wang, X.; Lv, Y.; Huang, D.; Wang, J.; Miao, R.; Nie, L.; Hao, J. Highly permeable thin film nanocomposite membrane utilizing a MoS2@NH2-UiO-66 interlayer for forward osmosis removal of Co2+, Sr2+ and Cs+ nuclide ions. *Appl. Surf. Sci.* **2023**, *611*, 155618. [CrossRef]
50. Yusaf, A.; Usman, M.; Ahmad, M.; Siddiq, M.; Mansha, A.; Al-Hussain, S.A.; Zaki, M.E.A.; Rehman, H.F. Highly Selective Methodology for Entrapment and Subsequent Removal of Cobalt (II) Ions under Optimized Conditions by Micellar-Enhanced Ultrafiltration. *Molecules* **2022**, *27*, 8332. [CrossRef] [PubMed]
51. Muhamad, N.; Makhtar, S.N.N.M.; Abdullah, N.; Pauzi, M.Z.M.; Mahpoz, N.M.; Othman, M.H.D.; Jaafar, J.; Abas, K.H.; Fadil, N.A.; Rahman, M.A. Composite zeolite hollow fiber membrane for the removal of nickel using forward osmosis. *J. Water Process Eng.* **2021**, *40*, 101806. [CrossRef]
52. He, M.; Wang, L.; Lv, Y.; Wang, X.; Zhu, J.; Zhang, Y.; Liu, T. Novel polydopamine/metal organic framework thin film nanocomposite forward osmosis membrane for salt rejection and heavy metal removal. *Chem. Eng. J.* **2020**, *389*, 124452. [CrossRef]
53. Qiu, F.; Chen, R.; Chung, T.-S.; Ge, Q. Forward osmosis for heavy metal removal: Multi-charged metallic complexes as draw solutes. *Desalination* **2022**, *539*, 115924. [CrossRef]
54. Kumar, J.; Joshi, H.; Malyan, S.K. Removal of Copper, Nickel, and Zinc Ions from an Aqueous Solution through Electrochemical and Nanofiltration Membrane Processes. *Appl. Sci.* **2021**, *12*, 280. [CrossRef]
55. Moradi, G.; Zinadini, S.; Rajabi, L.; Ashraf Derakhshan, A. Removal of heavy metal ions using a new high performance nanofiltration membrane modified with curcumin boehmite nanoparticles. *Chem. Eng. J.* **2020**, *390*, 124546. [CrossRef]
56. Lin, W.; Jing, L.; Zhang, B. Micellar-Enhanced Ultrafiltration to Remove Nickel Ions: A Response Surface Method and Artificial Neural Network Optimization. *Water* **2020**, *12*, 1269. [CrossRef]
57. Korus, I. Ultrafiltration enhanced with poly(sodium acrylate) as an effective method for separation of heavy metals from multicomponent solutions. *Desalin. Water Treat.* **2021**, *242*, 38–46. [CrossRef]
58. Abdulkarem, E.; Ibrahim, Y.; Naddeo, V.; Banat, F.; Hasan, S.W. Development of Polyethersulfone/α-Zirconium phosphate (PES/α-ZrP) flat-sheet nanocomposite ultrafiltration membranes. *Chem. Eng. Res. Des.* **2020**, *161*, 206–217. [CrossRef]
59. Wołowicz, A.; Staszak, K.; Hubicki, Z. Removal of Copper(II) in the Presence of Sodium Dodecylobenzene Sulfonate from Acidic Effluents Using Adsorption on Ion Exchangers and Micellar-Enhanced Ultrafiltration Methods. *Molecules* **2022**, *27*, 2430. [CrossRef] [PubMed]
60. Zheng, J.; Zhang, X.; Li, G.; Fei, G.; Jin, P.; Liu, Y.; Wouters, C.; Meir, G.; Li, Y.; Van der Bruggen, B. Selective removal of heavy metals from saline water by nanofiltration. *Desalination* **2022**, *525*, 115380. [CrossRef]
61. Hamid, M.F.; Abdullah, N.; Yusof, N.; Ismail, N.M.; Ismail, A.F.; Salleh, W.N.W.; Jaafar, J.; Aziz, F.; Lau, W.J. Effects of surface charge of thin-film composite membrane on copper (II) ion removal by using nanofiltration and forward osmosis process. *J. Water Process Eng.* **2020**, *33*, 101032. [CrossRef]
62. Abdullah, W.N.A.S.; Tiandee, S.; Lau, W.; Aziz, F.; Ismail, A.F. Potential use of nanofiltration like-forward osmosis membranes for copper ion removal. *Chinese J. Chem. Eng.* **2020**, *28*, 420–428. [CrossRef]
63. Hamid, M.F.; Abdullah, N.; Yusof, N.; Lau, W.J.; Ismail, A.F.; Wan Salleh, W.N.; Jaafar, J.; Aziz, F. Innovative polymer-complex draw solution for copper(II) removal using forward osmosis. *J. Environ. Chem. Eng.* **2021**, *9*, 104854. [CrossRef]
64. Harharah, R.H.; Abdalla, G.M.T.; Elkhaleefa, A.; Shigidi, I.; Harharah, H.N. A Study of Copper (II) Ions Removal by Reverse Osmosis under Various Operating Conditions. *Separations* **2022**, *9*, 155. [CrossRef]
65. Siddique, J.A. Nanofiltration membrane use for separation of heavy metals from wastewater. In *Emerging Techniques for Treatment of Toxic Metals from Wastewater*; Elsevier: Amsterdam, The Netherlands, 2023; pp. 523–549. ISBN 9780128228807.
66. Kahloul, M.; Mahfoudhi, S.; Ounifi, I.; Elabed, B.; Amor, T.B.; Hafiane, A. Green complexation for heavy metals removal from wastewater by Keggin-polyoxometalates enhanced ultrafiltration. *Water Sci. Technol.* **2022**, *86*, 1510–1526. [CrossRef] [PubMed]
67. Hoang, M.T.; Pham, T.D.; Verheyen, D.; Nguyen, M.K.; Pham, T.T.; Zhu, J.; Van der Bruggen, B. Fabrication of thin film nanocomposite nanofiltration membrane incorporated with cellulose nanocrystals for removal of Cu(II) and Pb(II). *Chem. Eng. Sci.* **2020**, *228*, 115998. [CrossRef]

68. Bandehali, S.; Parvizian, F.; Moghadassi, A.R.; Hosseini, S.M.; Shen, J.N. Fabrication of thin film-PEI nanofiltration membrane with promoted separation performances: Cr, Pb and Cu ions removal from water. *J. Polym. Res.* **2020**, *27*, 1–10. [CrossRef]
69. Cuhorka, J.; Wallace, E.; Mikulášek, P. Removal of micropollutants from water by commercially available nanofiltration membranes. *Sci. Total Environ.* **2020**, *720*, 137474. [CrossRef]
70. Saeedi-Jurkuyeh, A.; Jafari, A.J.; Kalantary, R.R.; Esrafili, A. A novel synthetic thin-film nanocomposite forward osmosis membrane modified by graphene oxide and polyethylene glycol for heavy metals removal from aqueous solutions. *React. Funct. Polym.* **2020**, *146*, 104397. [CrossRef]
71. Soo, K.W.; Wong, K.C.; Goh, P.S.; Ismail, A.F.; Othman, N. Efficient heavy metal removal by thin film nanocomposite forward osmosis membrane modified with geometrically different bimetallic oxide. *J. Water Process Eng.* **2020**, *38*, 101591. [CrossRef]
72. Ounifi, I.; Ursino, C.; Santoro, S.; Chekir, J.; Hafiane, A.; Figoli, A.; Ferjani, E. Cellulose acetate nanofiltration membranes for cadmium remediation. *J. Membr. Sci. Res.* **2020**, *6*, 226–234. [CrossRef]
73. Bhowmick, K.; Roy, S.; Mukherjee, M.; Sahoo, G.C.; Majumdar, S.; Mondal, P. Removal of cadmium by in-situ Cu nanoparticle enhanced ceramic-supported-polymeric composite NF membrane. *Mater. Today Proc.* **2021**, *47*, 1496–1499. [CrossRef]
74. Meng, Q.; Nan, J.; Mu, Y.; Zu, X.; Guo, M. Study on the treatment of sudden cadmium pollution in surface water by a polymer enhanced ultrafiltration process. *RSC Adv.* **2021**, *11*, 7405–7415. [CrossRef] [PubMed]
75. Can, M.; Arslan, F.; Çelik, M. Modelling of selective retention of Cd-Ni ions from aqueous solutions by polymer enhanced ultrafiltration. *Physicochem. Probl. Miner. Process.* **2022**, *58*, 151913. [CrossRef]
76. Yaqub, M.; Lee, S.H. Micellar enhanced ultrafiltration (MEUF) of mercury-contaminated wastewater: Experimental and artificial neural network modeling. *J. Water Process Eng.* **2020**, *33*, 101046. [CrossRef]
77. Yaqub, M.; Lee, S.H.; Lee, W. Investigating micellar-enhanced ultrafiltration (MEUF) of mercury and arsenic from aqueous solution using response surface methodology and gene expression programming. *Sep. Purif. Technol.* **2022**, *281*, 119880. [CrossRef]
78. Zhang, H.L.; Cai, H.; Xia, Y.; Zhang, P.; Xiong, S.W.; Gai, J.G. An l-cystine/l-cysteine impregnated nanofiltration membrane with the superior performance of an anchoring heavy metal in wastewater. *RSC Adv.* **2020**, *10*, 3438–3449. [CrossRef]
79. Han, D.S.; Solayman, K.M.D.; Shon, H.K.; Abdel-Wahab, A. Pyrite (FeS2)-supported ultrafiltration system for removal of mercury (II) from water. *Emergent Mater.* **2021**, *4*, 1441–1453. [CrossRef]
80. Ramírez-Rodríguez, L.C.; Díaz Barrera, L.E.; Quintanilla-Carvajal, M.X.; Mendoza-Castillo, D.I.; Bonilla-Petriciolet, A.; Jiménez-Junca, C. Preparation of a Hybrid Membrane from Whey Protein Fibrils and Activated Carbon to Remove Mercury and Chromium from Water. *Membranes* **2020**, *10*, 386. [CrossRef]
81. Abdullah, N.; Yusof, N.; Jye, L.W.; Jaafar, J.; Misdan, N.; Ismail, A.F. Removal of lead(II) by nanofiltration-ranged thin film nanocomposite membrane incorporated UiO-66-NH2: Comparative removal performance between hydraulic-driven and osmotic-driven membrane process. *J. Taiwan Inst. Chem. Eng.* **2021**, *128*, 354–369. [CrossRef]
82. Hanif, A.; Ali, S.; Hanif, M.A.; Rashid, U.; Bhatti, H.N.; Asghar, M.; Alsalme, A.; Giannakoudakis, D.A. A Novel Combined Treatment Process of Hybrid Biosorbent–Nanofiltration for Effective Pb(II) Removal from Wastewater. *Water* **2021**, *13*, 3316. [CrossRef]
83. Babaei, E.; Hashemifard, S.A. Polycarbonate/copper oxide mixed matrix membrane for separation of lead and cadmium from industrial effluents. *Sep. Sci. Technol.* **2022**, *57*, 619–636. [CrossRef]
84. Cao, D.-Q.; Wang, X.; Wang, Q.-H.; Fang, X.-M.; Jin, J.-Y.; Hao, X.-D.; Iritani, E.; Katagiri, N. Removal of heavy metal ions by ultrafiltration with recovery of extracellular polymer substances from excess sludge. *J. Memb. Sci.* **2020**, *606*, 118103. [CrossRef]
85. Zakmout, A.; Sadi, F.; Velizarov, S.; Crespo, J.G.; Portugal, C.A.M. Recovery of Cr(III) from Tannery Effluents by Diafiltration Using Chitosan Modified Membranes. *Water* **2021**, *13*, 2598. [CrossRef]
86. Zakmout, A.; Sadi, F.; Portugal, C.A.M.; Crespo, J.G.; Velizarov, S. Tannery Effluent Treatment by Nanofiltration, Reverse Osmosis and Chitosan Modified Membranes. *Membranes* **2020**, *10*, 378. [CrossRef]
87. Arif, Z.; Sethy, N.K.; Mishra, P.K.; Verma, B. Green approach for the synthesis of ultrafiltration photocatalytic membrane for tannery wastewater: Modeling and optimization. *Int. J. Environ. Sci. Technol.* **2020**, *17*, 3397–3410. [CrossRef]
88. Salman, R.H.; Hassan, H.A.; Abed, K.M.; Al-Alawy, A.F.; Tuama, D.A.; Hussein, K.M.; Jabir, H.A. Removal of chromium ions from a real wastewater of leather industry using electrocoagulation and reverse osmosis processes. *AIP Conf. Proc.* **2020**, *2213*, 020186.
89. Liu, Z.; Lei, M.; Chen, G.; Yuan, J. Treatment of Chromium Removal Wastewater from Tanning by a New Coupling Technology. *Processes* **2022**, *10*, 1134. [CrossRef]
90. Bratovcic, A.; Buksek, H.; Helix-Nielsen, C.; Petrinic, I. Concentrating hexavalent chromium electroplating wastewater for recovery and reuse by forward osmosis using underground brine as draw solution. *Chem. Eng. J.* **2022**, *431*, 133918. [CrossRef]
91. Engstler, R.; Reipert, J.; Karimi, S.; Vukušić, J.L.; Heinzler, F.; Davies, P.; Ulbricht, M.; Barbe, S. A Reverse Osmosis Process to Recover and Recycle Trivalent Chromium from Electroplating Wastewater. *Membranes* **2022**, *12*, 853. [CrossRef]
92. Pan, Z.; Yang, X.; Liang, Y.; Lyu, M.; Huang, Y.; Zhou, H.; Wen, G.; Yu, H.; He, J. Chromium-containing wastewater reclamation via forward osmosis with sewage sludge ash temperature-sensitive hydrogel as draw agent. *J. Water Process Eng.* **2023**, *51*, 103422. [CrossRef]
93. Shao, M.; Li, Y.; Meng, L.; Guo, J.; Gao, Y.; Liu, Y.; Huang, M. Simultaneous removal of antimony, chromium and aniline by forward osmosis membrane: Preparation, performance and mechanism. *Desalination* **2021**, *520*, 115363. [CrossRef]

94. Rashid, W.T.; Alkadira, I.A.; Jalhoom, M.G. Effect of Operating Conditions on Removal Heavy and Radioactive Elements by Reverse Osmosis Membrane. *Al-Qadisiyah J. Eng. Sci.* **2020**, *13*, 240–245. [CrossRef]
95. Samaei, S.M.; Gato-Trinidad, S.; Altaee, A. Performance evaluation of reverse osmosis process in the post-treatment of mining wastewaters: Case study of Costerfield mining operations, Victoria, Australia. *J. Water Process Eng.* **2020**, *34*, 101116. [CrossRef]
96. Lumami Kapepula, V.; García Alvarez, M.; Sang Sefidi, V.; Buleng Njoyim Tamungang, E.; Ndikumana, T.; Musibono, D.-D.; Van Der Bruggen, B.; Luis, P. Evaluation of Commercial Reverse Osmosis and Nanofiltration Membranes for the Removal of Heavy Metals from Surface Water in the Democratic Republic of Congo. *Clean Technol.* **2022**, *4*, 1300–1316. [CrossRef]
97. Japas, M.S.; Rubinstein, N.A.; Gómez, A.L.R. Revisiting reverse osmosis as a mechanism contributing to metal zoning in porphyry copper deposits. *Ore Geol. Rev.* **2022**, *143*, 104746. [CrossRef]
98. Pino, L.; Beltran, E.; Schwarz, A.; Ruiz, M.C.; Borquez, R. Optimization of nanofiltration for treatment of acid mine drainage and copper recovery by solvent extraction. *Hydrometallurgy* **2020**, *195*, 105361. [CrossRef]
99. Wang, C.; Sun, M.; Wang, X.; Zhang, X.; Zhao, Y.; Huo, M. Enhanced resource recovery from wastewater using electrochemical-osmotic system with nanofiltration membranes. *Resour. Conserv. Recycl.* **2022**, *186*, 106555. [CrossRef]
100. López, J.; Gibert, O.; Cortina, J.L. Evaluation of an extreme acid-resistant sulphonamide based nanofiltration membrane for the valorisation of copper acidic effluents. *Chem. Eng. J.* **2021**, *405*, 127015. [CrossRef]
101. Islam, M.S.; Vogler, R.J.; Abdullah Al Hasnine, S.M.; Hernandez, S.; Malekzadeh, N.; Hoelen, T.P.; Hatakeyama, E.S.; Bhattacharyya, D. Mercury removal from wastewater using cysteamine functionalized membranes. *ACS Omega* **2020**, *5*, 22255–22267. [CrossRef]
102. Ozbey-Unal, B.; Omwene, P.I.; Yagcioglu, M.; Balcik-Canbolat, Ç.; Karagunduz, A.; Keskinler, B.; Dizge, N. Treatment of organized industrial zone wastewater by microfiltration/reverse osmosis membrane process for water recovery: From lab to pilot scale. *J. Water Process Eng.* **2020**, *38*, 101646. [CrossRef]
103. Le, H.-S.; Qiu, Y.-R. Selective separation of Cd(II), Zn(II) and Pb(II) from Pb-Zn smelter wastewater via shear induced dissociation coupling with ultrafiltration. *Korean J. Chem. Eng.* **2020**, *37*, 784–791. [CrossRef]
104. Fu, J. Lead and Zinc Smelting Wastewater Treatment and Reclamation by Coagulation-Flocculation-Sedimentation, Ultrafiltration and Reverse Osmosis Technique. *J. Energy Environ. Chem. Eng.* **2021**, *6*, 94. [CrossRef]
105. Arabi, A.K.; Akram, B.; Mirbagheri, S.A. Industrial wastewater treatment by combining two systems of adsorption column and reverse osmosis. *J. Environ. Eng. Sci.* **2022**, *17*, 131–138. [CrossRef]
106. Hughes, A.E.; Haque, N.; Northey, S.A.; Giddey, S. Platinum Group Metals: A Review of Resources, Production and Usage with a Focus on Catalysts. *Resources* **2021**, *10*, 93. [CrossRef]
107. Crundwell, F.K.; Moats, M.S.; Ramachandran, V.; Robinson, T.G.; Davenport, W.G. Platinum-Group Metals, Production, Use and Extraction Costs. In *Extractive Metallurgy of Nickel, Cobalt and Platinum Group Metals*; Crundwell, F.K., Moats, M.S., Ramachandran, V., Eds.; Elsevier: Amsterdam, The Netherlands, 2011; pp. 395–409. [CrossRef]
108. Rao, C.R.M.; Reddi, G.S. Platinum group metals (PGM); occurrence, use and recent trends in their determination. *TrAC Trends Anal. Chem.* **2000**, *19*, 565–586. [CrossRef]
109. Mudd, G.M. Sustainability reporting and the platinum group metals: A global mining industry leader? *Platin. Met. Rev.* **2012**, *56*, 2–19. [CrossRef]
110. Ericsson, M.; Tegen, A. Global PGM mining during 40 years—A stable corporate landscape of oligopolistic control. *Miner. Econ.* **2016**, *29*, 29–36. [CrossRef]
111. Taghvaie Nakhjiri, A.; Sanaeepur, H.; Ebadi Amooghin, A.; Shirazi, M.M.A. Recovery of precious metals from industrial wastewater towards resource recovery and environmental sustainability: A critical review. *Desalination* **2022**, *527*, 115510. [CrossRef]
112. Swain, P.; Mallika, C.; Srinivasan, R.; Mudali, U.K.; Natarajan, R. Separation and recovery of ruthenium: A review. *J. Radioanal. Nucl. Chem.* **2013**, *298*, 781–796. [CrossRef]
113. Noah, N.F.M.; Othman, N.; Jusoh, N. Highly selective transport of palladium from electroplating wastewater using emulsion liquid membrane process. *J. Taiwan Inst. Chem. Eng.* **2016**, *64*, 134–141. [CrossRef]
114. Mohdee, V.; Ramakul, P.; Phatanasri, S.; Pancharoen, U. A numerical and experimental investigation on the selective separation of Pd (II) from wastewater using Aliquat 336 via hollow fiber supported liquid membrane. *J. Environ. Chem. Eng.* **2020**, *8*, 104234. [CrossRef]
115. Loreti, M.A.P.; Reis, M.T.A.; Ismael, M.R.C.; Staszak, K.; Wieszczycka, K. Effective Pd(II) carriers for classical extraction and pseudo-emulsion system. *Sep. Purif. Technol.* **2021**, *265*, 118509. [CrossRef]
116. Hanada, T.; Firmansyah, M.L.; Yoshida, W.; Kubota, F.; Kolev, S.D.; Goto, M. Transport of Rhodium(III) from Chloride Media across a Polymer Inclusion Membrane Containing an Ionic Liquid Metal Ion Carrier. *ACS Omega* **2020**, *5*, 12989–12995. [CrossRef] [PubMed]
117. Ma, Y.; Zeng, J.; Zeng, Y.; Zhou, H.; Liu, G.; Liu, Y.; Zeng, L.; Jian, J.; Yuan, Z. Preparation and performance of poly(4-vinylpyridine)-b-polysulfone-b-poly(4-vinylpyridine) triblock copolymer/polysulfone blend membrane for separation of palladium (II) from electroplating wastewaters. *J. Hazard. Mater.* **2020**, *384*, 121277. [CrossRef]
118. Jaafar, J.; Nasir, A.M. Grand Challenge in Membrane Fabrication: Membrane Science and Technology. *Front. Membr. Sci. Technol.* **2022**, *1*, 3. [CrossRef]

119. Wang, L.; Rehman, D.; Sun, P.-F.; Deshmukh, A.; Zhang, L.; Han, Q.; Yang, Z.; Wang, Z.; Park, H.-D.; Lienhard, J.H.; et al. Novel Positively Charged Metal-Coordinated Nanofiltration Membrane for Lithium Recovery. *ACS Appl. Mater. Interfaces* **2021**, *13*, 16906–16915. [CrossRef]
120. Purushothaman, M.; Harikrishnan, A.; Senthil Kumar, P.; George, J.; Rangasamy, G.; Vaidyanathan, V.K. Enhancement of antifouling properties, metal ions and protein separation of poly(ether-ether-sulfone) ultrafiltration membranes by incorporation of poly ethylene glycol and n-ZnO. *Environ. Res.* **2023**, *216*, 114696. [CrossRef]
121. Rahighi, R.; Hosseini-Hosseinabad, S.M.; Zeraati, A.S.; Suwaileh, W.; Norouzi, A.; Panahi, M.; Gholipour, S.; Karaman, C.; Akhavan, O.; Khollari, M.A.R.; et al. Two-dimensional materials in enhancement of membrane-based lithium recovery from metallic-ions-rich wastewaters: A review. *Desalination* **2022**, *543*, 116096. [CrossRef]
122. Chadha, U.; Selvaraj, S.K.; Vishak Thanu, S.; Cholapadath, V.; Abraham, A.M.; Zaiyan, M.; Manikandan, M.; Paramasivam, V. A review of the function of using carbon nanomaterials in membrane filtration for contaminant removal from wastewater. *Mater. Res. Express* **2022**, *9*, 012003. [CrossRef]
123. Huang, X.; Tian, F.; Chen, G.; Wang, F.; Weng, R.; Xi, B. Preparation and Characterization of Regenerated Cellulose Membrane Blended with ZrO2 Nanoparticles. *Membranes* **2021**, *12*, 42. [CrossRef]
124. Kwon, O.; Choi, Y.; Kang, J.; Kim, J.H.; Choi, E.; Woo, Y.C.; Kim, D.W. A comprehensive review of MXene-based water-treatment membranes and technologies: Recent progress and perspectives. *Desalination* **2022**, *522*, 115448. [CrossRef]

Disclaimer/Publisher's Note: The statements, opinions and data contained in all publications are solely those of the individual author(s) and contributor(s) and not of MDPI and/or the editor(s). MDPI and/or the editor(s) disclaim responsibility for any injury to people or property resulting from any ideas, methods, instructions or products referred to in the content.
